# Zebrafish arterial valve development occurs through direct differentiation of second heart field progenitors

**DOI:** 10.1093/cvr/cvae230

**Published:** 2024-10-26

**Authors:** Christopher J Derrick, Lorraine Eley, Ahlam Alqahtani, Deborah J Henderson, Bill Chaudhry

**Affiliations:** International Centre for Life, Biosciences Institute, Newcastle University, Central Parkway, Newcastle upon Tyne NE1 3BZ, UK; International Centre for Life, Biosciences Institute, Newcastle University, Central Parkway, Newcastle upon Tyne NE1 3BZ, UK; International Centre for Life, Biosciences Institute, Newcastle University, Central Parkway, Newcastle upon Tyne NE1 3BZ, UK; International Centre for Life, Biosciences Institute, Newcastle University, Central Parkway, Newcastle upon Tyne NE1 3BZ, UK; International Centre for Life, Biosciences Institute, Newcastle University, Central Parkway, Newcastle upon Tyne NE1 3BZ, UK

**Keywords:** Zebrafish, Heart development, Second heart field, Arterial valve, Bicuspid aortic valve

## Abstract

**Aims:**

Bicuspid aortic valve (BAV) is the most common congenital heart defect, affecting at least 2% of the population. The embryonic origins of BAV remain poorly understood, with few assays for validating patient variants, limiting the identification of causative genes for BAV. In both human and mouse, the left and right leaflets of the arterial valves arise from the outflow tract cushions, with interstitial cells originating from neural crest cells and the overlying endocardium through endothelial-to-mesenchymal transition (EndoMT). In contrast, an EndoMT-independent mechanism of direct differentiation of cardiac progenitors from the second heart field (SHF) is responsible for the formation of the anterior and posterior leaflets. Defects in either of these developmental mechanisms can result in BAV. Although zebrafish have been suggested as a model for human variant testing, their naturally bicuspid arterial valve has not been considered suitable for understanding human arterial valve development. Here, we have set out to investigate to what extent the processes involved in arterial valve development are conserved in zebrafish and, ultimately, whether functional testing of BAV variants could be carried out.

**Methods and results:**

Using a combination of live imaging, immunohistochemistry, and Cre-mediated lineage tracing, we show that the zebrafish arterial valve primordia develop directly from SHF progenitors with no contribution from EndoMT or neural crest, in keeping with the human and mouse anterior and posterior leaflets. Moreover, once formed, these primordia share common subsequent developmental events with all three aortic valve leaflets.

**Conclusion:**

Our work highlights a conserved ancestral mechanism of arterial valve leaflet formation from the SHF and identifies that development of the arterial valve is distinct from that of the atrioventricular valve in zebrafish. Crucially, this confirms the utility of zebrafish for understanding the development of specific BAV subtypes and arterial valve dysplasia, offering potential for high-throughput variant testing.

## Graphical Abstract

**Graphical Abstract:**
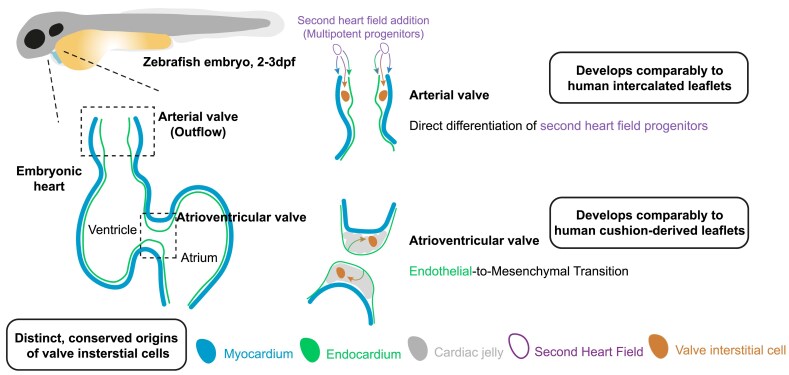

**Time of primary review: 4 days**

**See the editorial comment for this article ‘On the cusps of the second heart field: insights from zebrafish into arterial valve origins and disease ’, by R.G. Kelly, https://doi.org/10.1093/cvr/cvae249.**

## 1. Introduction

The aortic and pulmonary valves ensure unidirectional blood flow from the left and right ventricles to the systemic and pulmonary circulations, respectively. These valves can be impacted by congenital malformations, most frequently bicuspid aortic valve (BAV), which occurs in at least 2% of the population, typified by the presence of two valve leaflets rather than three.^1,2^ BAV can be present in isolation, but is also associated with other congenital heart malformations such as hypoplastic left heart syndrome^3^ or chromosomal disorders such as Down’s syndrome.^4^ Although frequently undetected at birth, BAV predisposes individuals to a spectrum of progressive aortic abnormalities, as well as degeneration of the valve with age, leading to stenosis or regurgitation and ultimately ventricular failure if left untreated.^5^ Defects of the pulmonary valve are less common^6^ but are strongly linked with different heart malformations such as tetralogy of Fallot.^3,7^

The semi-lunar valves develop in the arterial roots, located at the boundary between the ventricular myocardium and the smooth muscle of their respective arterial trunks, which in amniotes has a characteristic structure: hinge points attaching the leaflets to the myocardial wall, sinuses, and fibrous interleaflet triangles.^8,9^ Leaflet structure is broadly conserved across vertebrates, with apparently distinct elastin- and collagen-rich layers.^10,11^ Despite the importance of the arterial valves, remarkably little is known about the developmental mechanisms that give rise to their leaflets, and until recently, studies of the atrioventricular valves in multiple models had been extrapolated to understand arterial valve development.^12,13^

Addition of multipotent cardiac progenitors from the second heart field (SHF) is critical for normal arterial pole development.^14,15^ These cells express the transcription factor Islet1 (Isl1), which is down-regulated upon terminal differentiation following addition to the heart. At the arterial pole, the first wave of SHF cells forms cardiomyocytes^16,17^ and later smooth muscle.^8,18^ Within the distal outflow tract (OFT), cells pass through the transition zone where they co-express Isl1 and mature cardiomyocyte markers; this region eventually forms the arterial root.^14,15^ At embryonic day (E) 10.5-E11.5 in mice (Carnegie stage (CS) 14–16 in humans), two tongues of Isl1+ cells, which neither express myocardial nor smooth muscle markers, can be identified in the wall of the still unseptated OFT.^16,19,20^ The most proximal of these undifferentiated cells forms the intercalated valve swellings (ICVSs),^16,19,20^ and the remainder differentiate later into the smooth muscle of the arterial walls. Concomitant with ICVS formation, the two cardiac jelly-rich OFT cushions, which are found along the entirety of the (proximal) myocardial part of the OFT, are populated by cells from the overlying endocardium (itself SHF derived) through endothelial-to-mesenchymal transition (EndoMT) and also from the cardiac neural crest.^19–21^ These cushions expand and ultimately fuse, leading to OFT septation.^22,23^ The distal ends of these main cushions form the right and left leaflets of the aortic and pulmonary valve, whereas the ICVSs form the anterior and posterior/non-coronary leaflets.^19^ The different origins of these leaflets are reflected in the cell lineages they contain, as shown by Cre-based lineage tracing performed in mouse.^17^ All mesenchymal cells within these six primordia express the transcription factor Sox9.^20,24^ Notably however, the cells in the ICVSs differentiate directly from Isl1+ SHF progenitors into Sox9-expressing valve interstitial cells (VICs), where these cells co-express both markers for a short period.^19^ This is not seen in the OFT cushion-derived leaflets. A similar pattern of co-expression of ISL1 and SOX9 is visible in the developing ICVSs of human embryos between CS13-16, supporting this is a conserved mechanism of primordia formation.^20^ Despite these distinct origins, once established, all primordia undergo the same remodelling processes. The understanding of these distinct mechanisms of primordia formation may reflect the relationship between BAV subtypes^1,25^ and aortopathies.^1^ Therefore, there is need for an understanding of the relationship between OFT development, patient mutations, and their associated disease to identify the developmental origins of arterial pole malformations such as BAV.

Animal models have been invaluable in understanding the complex processes of valve formation, traditionally mouse, and more recently zebrafish. The rapid, temporally reproducible development of zebrafish is highly amenable to *in vivo* live imaging and, together with a high level of conservation of both process and gene regulatory networks, has made them a powerful model to investigate aspects of cardiac development not possible in mouse.^26^ The zebrafish has a single, naturally bicuspid arterial valve, residing in the unseptated OFT between the single ventricle and the bulbus arteriosus.^27,28^ This has led many to assume that it cannot be used to model human arterial valve development or disease. However, the bulbus arteriosus is a smooth muscle, elastin-rich structure,^28^ and both the bulbus arteriosus and distal part of the ventricle are derived from the SHF,^15,29^ resembling the amniote arterial root.^8^ In the few studies that have characterized the arterial valve in zebrafish,^30–33^ the origin of the leaflets and whether they have any similarities to human arterial valve development have not been investigated. Furthermore, similar to mouse, many others have made assumptions as to the similarity of arterial valve development with the atrioventricular valve.^12,34^ Establishing the mechanism by which the arterial valve forms, if similar to mouse/human, would enable zebrafish to be a powerful model for investigating key aspects of arterial pole development and disease.

In this study, we asked to what extent the processes involved in arterial valve development are conserved in zebrafish. We investigated whether the zebrafish arterial root and leaflets are structurally similar to those in the mouse/human, whether the zebrafish OFT possesses a transition zone, and if this is the site of arterial valve formation. We then examined whether the zebrafish arterial valve primordia develop through direct differentiation of SHF progenitors or by cellularization of an extracellular matrix (ECM)–rich cushion. By identifying the conserved developmental mechanisms leading to the formation of the zebrafish arterial valve primordia, we establish the suitability of zebrafish for understanding the development of specific BAV subtypes and arterial valve dysplasia and as a tool for patient variant analysis.

## 2. Methods

### 2.1 Zebrafish husbandry

Adult zebrafish (*Danio rerio*) were maintained according to standard laboratory conditions and all procedures carried out in accordance with the local Animal Welfare and Ethical Review Body (AWERB), UK Home Office and Newcastle University (Project Licences P25F4F0F4 and PP0696166), in line with Directive 2010/63/EU of the European Parliament. Adult zebrafish were euthanized by terminal anaesthesia using tricaine methanesulfonate (MS222, Merck E10521) followed by destruction of the brain. Details of specific lines used and protocols for collection of embryonic and adult tissues are listed in the Supplementary material online, *Method S1*.

### 2.2 Mouse tissue

WT C57BL/6 mice were maintained according to standard laboratory conditions and all procedures carried out in accordance with the local Animal Welfare and Ethical Review Body (AWERB) and Newcastle University (Project Licences 30/3876 and P9E095FF4) in line with Directive 2010/63/EU of the European Parliament. At P90, the animal was culled by cervical dislocation, the heart excised, and the region of interest dissected, fixed in 4% PFA, serial dehydrated, and embedded in paraffin wax (VWR, 361077E) following standard protocols. Wax sections were cut at 8 μm thick using a Leica RM2235 Microtome, with alternating sections split between three different groups for analysis of elastin, proteoglycans and collagen within the same heart.

### 2.3 Live imaging

*Tg(kdrl:GFP)* embryos were imaged in 2% methyl cellulose (Sigma M0262) on an Olympus BX61 microscope using cellSens Dimension software (Evident).

### 2.4 DAF-FM staining

DAF-FM (Thermo, D23842) was diluted in DMSO (Thermo, 11385597) at a stock concentration of 5 mM. Thirty minutes prior to fixation, 20 manually dechorionated embryos were incubated in 3 mL of 5 μM DAF-FM in E3 at 28.5°C; the final concentration of DMSO in E3 was 1%. Embryos were fixed in 4% PFA overnight at 4°C, then dehydrated to 100% MeOH, and stored at −20°C. Embryos were protected from the light during incubation and prior to immunohistochemistry.

### 2.5 BrdU incorporation

BrdU (Merck, B5002) was dissolved in E3 at a concentration of 10 mg/mL, filtered using a 0.22 μm filter, aliquoted, and stored at −20°C long term. For BrdU pulses, 20 manually dechorionated *Tg(kdrl:GFP)* embryos were incubated in 3 mL of 5 mg/mL BrdU in E3 at 28.5°C. Embryos were rinsed in E3 and fixed in 4% PFA overnight at 4C, then dehydrated to 100% MeOH, and stored at −20°C.

Following rehydration and prior to immunohistochemistry, embryos were incubated in 2N HCl for 1 h at room temperature and were then extensively washed prior to blocking (see Section 2.8).

### 2.6 Paraffin embedding of adult zebrafish tissue

For Miller’s elastin, alcian blue, and Masson’s trichrome, adult hearts were washed from 70% EtOH into 100% EtOH and left in 100% EtOH overnight. The next day, hearts were incubated in 50-50 100% EtOH-xylene at room temperature for 30 min, followed by two washes in 100% xylene at 65°C, 1 h in 50-50 xylene-wax at 65°C, two incubations in wax at 65°C for 30 min, and a final 45 min incubation in wax at 65°C, before embedding in wax. Wax sections were cut at 8 μm thick, with alternating sections split between three different groups for analysis of elastin, proteoglycans, and collagen within the same heart.

For whole adult histology, embryos were cut to remove the trunk and washed from 70% EtOH into 100% EtOH and left in 100% EtOH overnight. The next day, hearts were incubated in xylene at room temperature for 30 min twice, followed by 1 h in 50-50 xylene-wax at 65°C and four incubations in wax at 65°C for 1 h, before embedding in wax. Wax sections were cut at 8 μm thick and stained with haematoxylin (Harris, Sigma HHS16) and 10% Eosin Y (Thermo, 6766010) using standard protocols.

### 2.7 ECM stains on paraffin sections

Wax sections were cleared in xylene and rehydrated to MQ H_2_O. To stain for elastin, slides were incubated in Miller’s elastin (VWR, 351154S) for 2 h (mouse) or 1 h (zebrafish), rinsed in ddH_2_O, incubated in fresh 3% Iron (III) Chloride for 20 min (mouse) or 10 min (zebrafish) (Merck, 157740), rinsed in running tap water, and counterstained in 10% Eosin Y for 5 min. Alcian blue staining (Abcam AB150662) was carried out according to the manufacturer’s instructions. Prior to collagen staining, rehydrated sections were incubated in Bouin’s solution (Sigma HT10132) for 16 h overnight at room temperature washed under running tap water for 8 min and rinsed for 2 min in ddH_2_O. Slides were incubated in Weigert’s Iron haematoxylin (Sigma HT1079) for 7 min and rinsed under running tap water for 3 min and ddH_2_O for 2 min. Using Masson’s Trichrome kit (Sigma, H15-1KT), slides were then incubated in Biebrich Scarlet-Acid Fuchsin for 7 min, rinsed in ddH_2_O for 2 min, and incubated in Phosphotungstic/Phosphomolybdic acid for 45 min (with agitation), aniline blue for 45 min (with agitation), and fresh 1% acetic acid for 2 min with three quick rinses in ddH_2_O. Once stained, all slides were cleared in xylene, dehydrated and mounted in histomount (National Diagnostics HS-103).

### 2.8 Immunohistochemistry on zebrafish embryos

Embryos were rehydrated from 100% MeOH to PBST and rinsed in PBS-X (1× PBS with 0.2% Triton-X (Sigma T8787)) and then blocked for 1 h at room temperature in 5% normal goat serum (Fisher Scientific 10098792) and 10 mg/mL bovine serum albumin (A2153, Sigma) in PBS-X (blocking buffer). The following primary antibodies were used: cardiac troponin (DSHB CT3, Mouse IgG2a, 1:200), Tagln (Stratech GTX125994, Rabbit, 1:1000), BrdU (Abcam ab6326, Rat, 1:200), GFP (Abcam 13970, Chick, only used for BrdU and FISH, 1:500), DsRed (also binds mCherry, Takara Bio 632496, Rabbit, 1:200), Isl1/2 (DSHB 39.4D5, Mouse IgG2b, 1:50), MLCK (Merck SAB4200808 (clone K36), Mouse IgG2b, 1:200), and Sox9 (Abcam ab185230, Rabbit, 1:500). Primary antibodies were incubated in blocking buffer and 1% DMSO (Thermo, 042780-AK) at 4°C overnight with agitation. The next day, embryos were rinsed in PBS-X and washed thoroughly over 2 h at room temperature. The following secondary antibodies were used (Thermo): Donkey anti-Mouse 647 (A31571), Goat anti-Rabbit 594 (A11012), Donkey anti-Rat 594 (A21209), Goat anti-Chick 488 (A11039), Donkey anti-Rabbit 568 (A11042), Goat anti-Mouse IgG2a 350 (A21130), Goat anti-Mouse IgG2b 594 (A22145), Donkey anti-Rabbit 647 (A31573), Goat anti-Mouse IgG2a 647 (A21241), Goat anti-Mouse IgG2a 488 (A21131), and Goat anti-Mouse IgG2b 647 (A21242). Secondary antibodies were incubated in blocking buffer at 1:200 dilution with 1% DMSO at 4°C overnight with agitation. The next day, embryos were rinsed three times in PBS-X and rapidly dehydrated to 100% EtOH for resin embedding, which is described in the Supplementary material online, *Method S1*.

### 2.9 Generation of *elnb* probe

The *elnb* (ENSDARG00000069017) probe was cloned using primers forward: 5′-ATTAGGGGCTGGTGTTGGAA-3′ and reverse: 5′-CCAAGTCCAAATCCAGCACC-3′ from 76 hpf WT (AB) cDNA. The 1089 bp fragment was ligated into pCRII-TOPO (Fisher 11503837) and sequenced by Sanger sequencing (Eurofins) to confirm insertion. For antisense mRNA, the plasmid was linearized with BamHI (NEB R0136) and transcribed using T7 (Promega, P207E) in the presence of dioxygen-labelled nucleotides (Merck, 11277073910). mRNA *in situ* hybridization protocols are listed in the Supplementary material online, *Method S1*.

### 2.10 Miller’s elastin staining of embryonic zebrafish

Embryos were rehydrated to ddH_2_O and incubated in Miller’s elastin for 8 h at room temperature, rinsed in ddH_2_O, incubated in freshly made 3% Iron (III) Chloride for 1 h at room temperature, rinsed in ddH_2_O, and incubated in 10% Eosin Y overnight for 15 h at room temperature. The next day, embryos were rinsed in ddH_2_O and dehydrated to 100% EtOH for resin embedding.

### 2.11 Three-dimensional reconstructions

Serial sections of resin-embedded adult zebrafish hearts, cut at 7 μm thick, were stained with haematoxylin and eosin, photographed, and reconstructed using Amira 2020.2 (Thermo).

Immunohistochemistry, sectioning, and imaging of WT sibling and *vangl2* mutant embryos were carried out as described. DAPI and MLCK channels and *Tg(kdrl:GFP)* and cardiac troponin channels were combined in Fiji and converted to an RGB image. Both stacks were imported into Amira and aligned, ensuring that both stacks aligned with one another. Segmentation of the endocardium, lumen, myocardium, and smooth muscle was performed and the space remaining defined as either the sinistral or dextral primordia. Volume measurements were extracted from the Material Statistics feature.

### 2.12 Study design and statistical analysis

Each experiment was performed at least twice, composing of embryos from different clutches from different parents collected on different days. BrdU analysis was performed three times, with each experimental replicate represented by five embryos; in the same embryos, the number of cells was quantified in the primordia. Sample size was pre-determined before analysis. For all other analyses, individual embryos represent an experimental replicate and sample size was not pre-determined. H&E and Miller’s elastin on embryos (<5 dpf) were performed on both WT(AB) and Casper; *Tg(kdrl:GFP)* genotypes. No differences were observed. All statistical analyses were carried out in GraphPad Prism.

## 3. Results

### 3.1 Conservation of arterial valve structure in adult zebrafish

**Figure 1:**
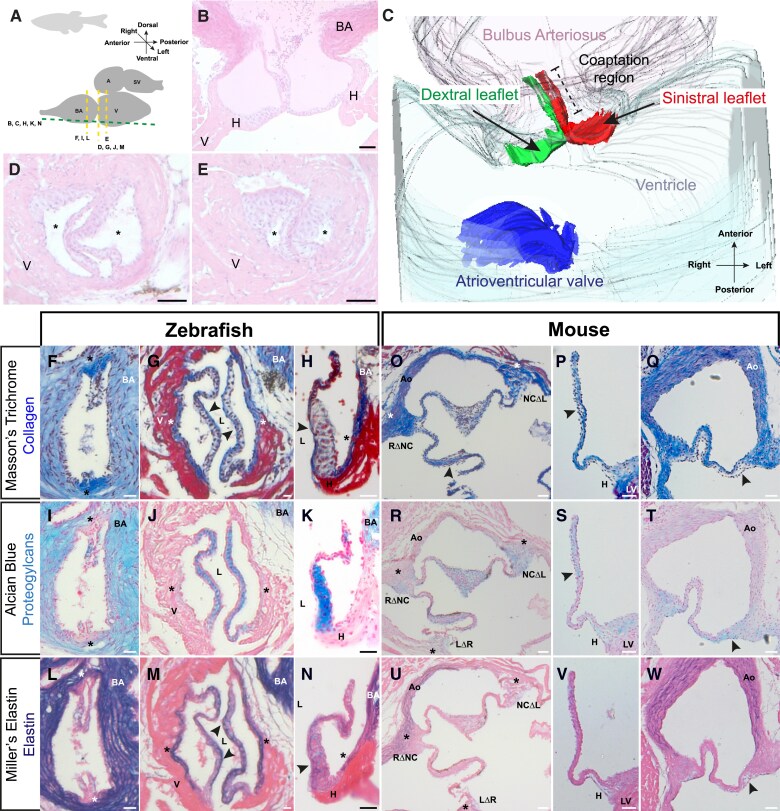
The zebrafish arterial valve is anatomically similar to other vertebrate arterial valves. (*A*) Overview of the orientation of the heart in adult zebrafish, with planes of imaging for C-N. (*B*) Haematoxylin and eosin–stained long-axis sections of excised adult zebrafish heart (female, 9 months old) (*n* = 4). (*C*) 3D reconstruction of the arterial pole, viewed ventrally, reconstructed from (*B*). The arterial valve leaflets (green, red) are hinged in the ventricular myocardium and span the myocardial (grey)-arterial (pink) boundary and are not in continuity with the atrioventricular valve (blue). The arterial valve leaflets are aligned along the anterior-posterior axis of the zebrafish and are defined by their position within the adult: dextral (green) and sinistral (red) with their coaptation region spanning the myocardial-arterial boundary. (*D* and *E*) Haematoxylin and eosin–stained short-axis sections of excised adult zebrafish heart (male, 9 months old) at the level of distal (*D*) and proximal myocardium (*E*) showing two leaflets of the arterial valve, with sinuses (asterisks) between the leaflets and the wall (*n* = 4). (*F*) Masson’s trichrome-stained short-axis section of excised adult zebrafish heart (female, 14 months) at the commissures of arterial leaflets in the bulbus arteriosus. Collagen (blue) is present in the bulbus arteriosus, enriched in the commissures and largely absent from the leaflets. (*G*) Same heart as (*F*) at the proximal myocardium. Collagen is present in the lumen facing portion of the leaflets (arrowheads), the wall of the sinus (asterisks) but is excluded from the leaflet interstitium. (*H*) Masson’s trichrome-stained long-axis section of excised adult zebrafish heart (female, 12 months old) showing the sinistral leaflet. Collagen is present on the luminal surface (arrowhead) of the leaflet, the root, and the bulbus arteriosus. (*I*) Alcian blue–stained sections, same heart shown in (*F*) and (*G*). Proteoglycans (blue) are abundant in the bulbus arteriosus and leaflets but absent from the commissures (asterisks). (*J*) Same heart as (*I*) at the proximal myocardium. Proteoglycans are present throughout the interstitium of the leaflets, with very weak signal in the sinus wall (asterisk). (*K*) Alcian blue–stained section, same heart shown in (*H*), showing the bulbus arteriosus and leaflets are rich in proteoglycans. (*L*) Miller’s elastin-stained sections, same heart shown in (*F*), (*G*), (*I*), and (*J*). Elastin (purple) is abundant in the bulbus arteriosus, absent from the tips of the leaflets and commissures (asterisks). (*M*) Same heart as (*L*) at the proximal myocardium. Elastin is present throughout the leaflet, but is enriched on the luminal surface of the leaflets and the sinus wall (arrowheads). (*N*) Miller’s elastin-stained section, same heart shown in (*H*) and (*K*). Elastin is present in the bulbus arteriosus, enriched in the lumen facing aspect of the arterial leaflets, and present in the root of the valve (for *F*, *G*, *I*, *J*, *L*, and *M*: *n* = 5; for *H*, *K*, and *N*: *n* = 4). (*O*) Masson’s trichrome-stained short-axis section of postnatal day (P) 90 mouse aortic valve. The aorta and commissures (asterisks) are rich in collagen, whilst there is enrichment on the sinus facing aspect of the leaflets (arrowhead). (*P* and *Q*) Masson’s trichrome-stained long-axis section P90 mouse aortic valve, showing left (*P*) and non-coronary leaflets (*Q*). Collagen is present in the aortic root, hinge, and wall. In the leaflets, collagen is mainly localized to the arterial aspect and absent from the luminal surface (*P* and *Q*, arrowheads). (*R*) Alcian blue–stained section of same heart shown in (*O*). Proteoglycans are present in the leaflets and commissures (asterisks) but absent from the aorta. (*S* and *T*) Alcian blue–stained section, same heart shown in (*P*) and (*Q*). Sulfated proteoglycans are present in the aortic root and hinge, but largely absent for the wall of the aorta. (*U*) Miller’s elastin-stained section, same heart shown in (*O*) and (*R*). Elastin fibres are present in the aorta and absent from the commissures (asterisks) and valve leaflets. (*V* and *W*) Miller’s elastin-stained section, same heart shown in (*P*), (*Q*), (*S*), and (*T*). Mature elastin fibres are present in the wall of the aorta, with diffuse staining in the hinge. There are no clear fibres of elastin in the leaflets. (*B*), (*H*), (*K*), (*N*), (*P*), (*Q*), (*S*), (*T*), (*V*), and (*W*): anterior: up, left: right. (*D*)–(*G*),( *I*), (*J*), (*L*), and (*M*): dorsal: up, left: right. V, ventricle; BA, bulbus arteriosus; A, atrium; SV, sinus venosus; LV, left ventricle; Ao, aorta; H, hinge; L, lumen; RΔNC, right/non-coronary commissure; NCΔL, non-coronary/left commissure; LΔR, left/right commissure. Scale bars: (*B*) and (*D*)–(*N*): 20 μm; (*O*)–(*W*): 50 μm.

We have previously characterized the morphology of the arterial roots in mice and human.^8,19,35^ For comparison, we performed three-dimensional reconstruction to extend descriptions of the adult zebrafish arterial valve^27,28,36^ (*Figure 1A–C*). As in amniotes, the zebrafish arterial valve is hinged at the distal extreme of the ventricle with the leaflets extending beyond the myocardial-smooth muscle boundary. The leaflets are aligned almost perpendicular to the left-right axis of the embryo and have a plane of apposition along the anterior-posterior axis of the embryo (*Figure 1B–E*; Supplementary material online, *Figure S1*). To avoid confusion with mouse/human arterial leaflets, we have defined these as the dextral and sinistral leaflets given their predominant anatomical position within the adult (*Figure 1B* and *C*; Supplementary material online, *Figure S1*). Overall, the two leaflets had no distinguishing morphological features, and there was no continuity between either leaflet and the atrioventricular valve (*Figure 1C*). Sections cut across the short axis of the OFT clearly identified sinuses between the leaflet and the wall (*Figure 1A*, *D*, and *E*, asterisks). The tips of the leaflets were particularly thin and often difficult to visualize in sections (*Figure 1B*), but evaluation in multiple planes suggested a broad coaptation region spanning the myocardial-arterial boundary (*Figure 1C*).

Adult human arterial valve leaflets are widely described to have a trilaminar arrangement of ECM: a fibrous layer of collagen on their arterial aspect, a proteoglycan-rich middle layer, and an elastin-rich layer on the ventricular/luminal side.^10,11^ With this in mind, we investigated the localization of these ECM components in the adult zebrafish arterial valve.

Masson’s trichrome staining revealed high levels of collagen in the bulbus arteriosus that is enriched at the commissures (the points of apposition of the leaflets close to the wall) (*Figure 1F* asterisk; Supplementary material online, *Figure S2A*). Low levels of collagen were found in the leaflet interstitium, with stronger staining on the luminal facing surface (*Figure 1G*, arrowhead) and sinus wall that overlies ventricular myocardium (*Figure 1H*, asterisk).

The whole of the bulbus arteriosus and leaflet interstitium stained strongly with alcian blue (*Figure 1I–K*; Supplementary material online, *Figure S2B*) indicating an abundance of sulphated proteoglycans, with no signal in the commissures (*Figure 1I*, asterisk), and very low levels were found in the sinus wall (*Figure 1J* asterisk; Supplementary material online, *Figure S2B*).

Mature elastin, identified by Miller’s stain, was absent from the commissures and tips of the leaflets (*Figure 1L*, asterisk), but was found throughout the leaflets proximally, with stronger staining on the luminal side (*Figure 1M* and *N*, arrowhead), overlapping with collagen (*Figure 1G*). Elastin was also present in the sinus walls (*Figure 1M* and *N*, arrowhead) although the hinges were deficient in elastin (*Figure 1N*; Supplementary material online, *Figure S2C*). The bulbus arteriosus stained strongly for elastin, although we did not detect discrete elastin fibres (*Figure 1L–N*; Supplementary material online, *Figure S2C*).

As most of the data about arterial valve formation has been gathered from studies using mouse, we compared the zebrafish arterial valve with mouse more closely, analysing aortic valves from 90-day-old adults (P90). In the P90 mouse aortic valve, collagen was present throughout the wall, commissures, and hinges, but was restricted to the arterial-facing aspect in all three leaflets (*Figure 1O–Q*, arrowheads; Supplementary material online, *Figure S2D* and *E*). As in the zebrafish leaflets, alcian blue staining was reciprocal to collagen, mostly present in the tips and hinge (*Figure 1R–T* asterisks; Supplementary material online, *Figure S2F* and *G*). In contrast to that observed in zebrafish, alcian blue was present in the commissures in mouse (*Figure 1R*), overlapping with collagen (*Figure 1O*). Furthermore, whilst the bulbus arteriosus was rich in sulfated proteoglycans, the mouse aorta stained very weakly with alcian blue (*Figure 1R* and *T*; Supplementary material online, *Figure S2B* and *F*).

As in zebrafish, the commissures in mouse were devoid of elastin (*Figure 1U*), but mature fibres were clearly visible in the aorta (*Figure 1U–W*; Supplementary material online, *Figure S2H* and *I*). Strikingly, we detected minimal elastin in the adult mouse aortic valve leaflets, with weak and sparse staining localized to the tips of the leaflets (*Figure 1U–W*; Supplementary material online, *Figure S2I*). Melanocytes were readily detectable in the mouse aortic valve leaflets (*Figure 1O*, *R*, and *U*; Supplementary material online, *Figure S2E*, *G*, and *I*), but not in the zebrafish arterial valve leaflets (*Figure 1B*, *D–H*, *I–K*, and *L–N*).

Together, these data show that whilst elastin content and collagen localization within arterial leaflets differs between zebrafish and mouse, there are clear and fundamental hallmarks of arterial valves. The arterial root is fibrous; the leaflets themselves are rich in sulfated proteoglycans that do not co-localize with collagen; commissures stain strongly for collagen and are devoid of elastin; and the arterial aspect of the outflow is rich in elastin and collagen. Taken together, these analyses indicate general structural similarity between the adult zebrafish arterial valve and the adult mouse aortic valve.

### 3.2 Embryonic development of the arterial valve in zebrafish follows conserved events

Arterial valve formation is a sequence of key events: establishment of primordia, sinus formation, and leaflet sculpting.^9,19,35^ To identify if these steps also occur in zebrafish, we examined histological sections of the developing arterial pole during embryogenesis.

**Figure 2:**
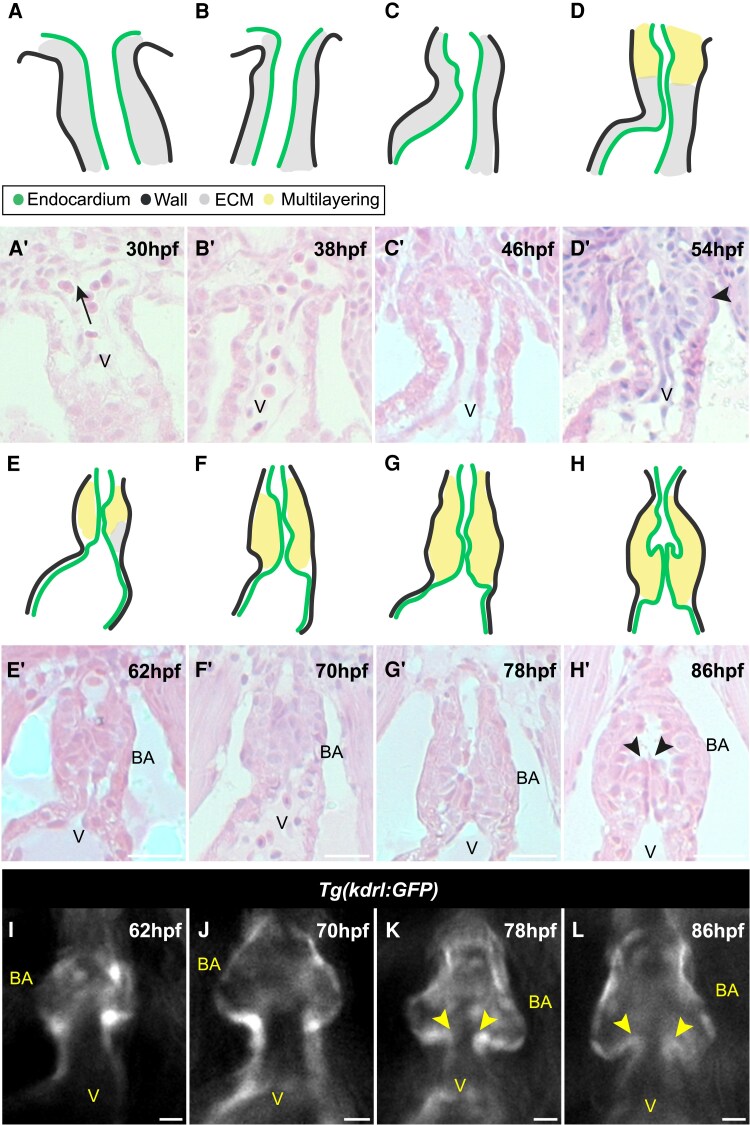
Development of the zebrafish arterial valve follows conserved events. (*A*) Schematic of 30 hpf OFT. Two cell layers, the wall (black) and endocardium (green), are separated by ECM (grey). (*A*′) Representative haematoxylin and eosin–stained midline section through the long axis of the OFT at 30 hpf; arrow denotes direction of blood flow (*n* = 6). (*B*–*C*′) Between 38 and 46 hpf, the two cell layers remain distinct and separated by ECM (38 hpf, *n* = 7; 46 hpf, *n* = 10). (*D* and *D*′) At 54 hpf, a multi-layering of cells is present at the distal-most point of the OFT where no ECM is visible (*D*′, arrowhead), with cells of unknown fate in yellow (*n* = 8). (*E* and *E*′) ECM in the ventricle and OFT is largely absent at 62 hpf (*n* = 11). (*F*–*G*′). Between 70 and 78 hpf, any remaining ECM is lost and the OFT lengthens and tapers where it connects to the ventral aorta (70 hpf, *n* = 5; 78 hpf, *n* = 10). (*H* and *H*′) Sinuses are visible by 86 hpf, defining two leaflets in the OFT (arrowheads) (*n* = 5/8). (*I–L*) Live fluorescent imaging of the OFT between 62 and 86 hpf of *Tg(kdrl:GFP)* embryos, marking the endocardium. Two primordia are visible at 62 hpf (*I*) (*n* = 13) and 70 hpf (*J*) (*n* = 14). The beginning of sinus formation is detectable at 78 hpf (*K*) (*n* = 11/15), with tips of leaflets visible (arrowheads) and is mostly complete by 86 hpf (*L*) (*n* = 17). V, ventricle; BA, bulbus arteriosus. Scale bars: (*A*)–(*H*): 20 μm; (*I*)–(*L*): 5 μm.

Initially, at 30 hpf, the embryonic OFT is a single outer layer of myocardium, with an inner layer of endocardium, separated by ECM (cardiac jelly)^37^*(Figure2A*–*B′*). At 46 hpf, the outflow has elongated through SHF addition at the distal end,^15,38^ with no evidence of any arterial valve structures (*Figure 2C* and *C′*). By 54 hpf, the clear outer layer-ECM endocardial layering is still observed in the proximal OFT (*Figure 2D* and *D′*). Distally, however, little or no ECM is visible (arrowhead, *Figure 2D′*), and a multi-layered arrangement of cells is apparent (*Figure 2D*, yellow), forming a bulge that narrows the lumen of the vessel, with the distinction between cell types lost. At 62 hpf, the bulge of cells in the distal OFT appears larger and the ECM in the proximal OFT and the ventricle has thinned (*Figure 2E–E′*). Between 70 and 78 hpf, the OFT noticeably lengthens with a tapering at the point of connection to the ventral aorta distally (*Figure2F*–*G′*). At 86 hpf, sinuses are visible between the bulge of cells and the outflow wall for the first time, delineating the two leaflets of the arterial valve. At the same time, the OFT takes on a more pear-shaped profile around the forming valve (*Figure 2H* and *H′*). Over the remaining course of embryonic development (86–118 hpf), there is little change in the appearance of the arterial valve leaflets (see Supplementary material online, *Figure S3A–D*), although they have thinned by 14 dpf (see Supplementary material online, *Figure S3E*), indicating that sculpting happens during post-embryonic stages.

We confirmed the window of primordia formation by quantifying the distance between the wall of the heart and the endocardium in *Tg(kdrl:GFP)* embryos in which all endothelial cells, including the endocardium, express GFP (see Supplementary material online, *Figure S3F*). At 46 hpf, wall thickness in the distal OFT was uniform (see Supplementary material online, *Figure S3G–G″*), whilst at 54–70 hpf, we identified an increased wall thickness, coinciding with the multi-layered appearance of the distal OFT (see Supplementary material online, *Figure S3H–J″*). Thus, the bulge of cells that is seen in the distal OFT from 54 hpf (*Figure 2D′*) is the primordia of the arterial valve leaflets.

To extend and complement our histological analysis, we performed live imaging of the developing OFT to identify movement of valve leaflets. In *Tg(kdrl:GFP)* embryos, the valve primordia could be visualized at 62–70 hpf, but no leaflets were present (*Figure 2I* and *J*; Supplementary material online, *Movies S1* and *S2*). By 78 hpf, rudimentary leaflets and sinuses could be distinguished, although the extent of their demarcation varied between embryos within the same clutch (*Figure 2K*; Supplementary material online, *Movie S3*). By 86 hpf, two leaflets, each with a distinct sinus, were identified in all embryos (*Figure 2L*; Supplementary material online, *Movie S4*).

In summary, the zebrafish OFT progresses through the conserved, landmark events of arterial valve formation, from an initially open, continuous vessel (30–46 hpf), through primordia establishment (46–70 hpf), sinus formation (70–86 hpf), and finally leaflet sculpting (86 hpf onwards).

### 3.3 Arterial valve primordia form at the transition zone

**Figure 3:**
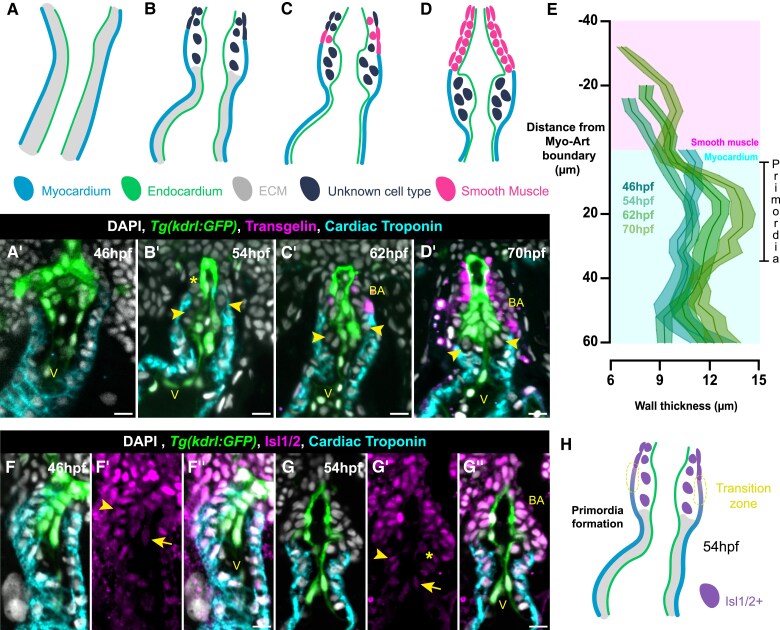
Arterial valve primordia form at the transition zone. (*A–D*) Schematics of OFT at 46 hpf (*A*), 54 hpf (*B*), 62 hpf (*C*), and 70 hpf (*D*). (*A*′–*D*′) Representative midline sections of *Tg(kdrl:GFP)* (endocardium, green) embryos stained for Tagln (smooth muscle, magenta), cardiac troponin (myocardium, cyan), and DAPI (white) between 46 and 70 hpf. At 46 hpf (*A* and *A*′), the entire OFT is myocardial (*n* = 12); at 54 hpf (*B* and *B*′), there is a region distally that is not myocardial and does not express Tagln (asterisk); some cells are identifiable between the endocardium and myocardium (arrowheads) (*n* = 15). By 62 hpf (*C* and *C*′), the region distal to the myocardium begins to express Tagln (*n* = 9), which is more robust; at 70 hpf (*D* and *D*′) (*n* = 11), this is the bulbus arteriosus. Cells present between the endocardium and myocardium do not express any markers (*C*′ and *D*′ arrowheads). (*E*) Quantification of wall thickness, averaged across left and right, with distance along OFT measured relative to myocardial-arterial boundary (46 hpf, *n* = 24; 54 hpf, *n* = 30; 62 hpf, *n* = 18; 70 hpf, *n* = 22). The primordia of the arterial valve form at the distal-most point of the ventricular myocardium. (*F*–*G*″) Representative midline resin section of *Tg(kdrl:GFP)* (green), stained for cardiac troponin (cyan), Isl1/2+ cells (SHF, magenta), and DAPI (white) at 46 hpf (*F*–*F*″, *n* = 14) and 54 hpf (*G*–*G*″, *n* = 14). (*F*–*F*″) At 46 hpf, both cardiomyocytes (arrowhead) and endocardium (arrow) at the distal end of the OFT are Isl1/2+ (*n* = 14). (*G*–*G*″) At 54 hpf, cardiomyocytes (arrowhead) and endocardium (arrow) of the distal OFT are Isl1/2+, whilst cells of the forming primordia are Isl1/2 (asterisk), but do not express myocardial or endocardial markers. The forming bulbus arteriosus is also SHF derived. (*H*) Summary of expression data, confirming the presence of the transition zone at the arterial pole (yellow region) as the site of primordia formation. E, mean ± SEM; V, ventricle; BA, bulbus arteriosus. Scale bars: 10 μm.

To identify the position at which the arterial valve primordia form in zebrafish, we performed immunohistochemistry for cardiac troponin (myocardium) and transgelin (Tagln; formerly SM22α), a well-conserved marker of smooth muscle cells in the arterial pole^8,19,39^ in *Tg(kdrl:GFP)* embryos. At 46 hpf, when there were no primordia present (*Figure 2*; Supplementary material online, *Figure S3*), the entire OFT wall was myocardial (*Figure 3A–A′*). At 54 hpf, the beginning of primordia formation, we could identify distally positioned cells that did not express troponin nor Tagln (*Figure 3B* and *B′*, asterisk). By 62 hpf, cells in the wall distal to the valve primordia were sparsely Tagln+ (*Figure 3C* and *C′*), and by 70 hpf, Tagln expression was robust in the distal OFT (*Figure 3D* and *D′*). From 54 hpf, the arterial valve primordia were clearly seen within the myocardial portion of the OFT at the boundary of cardiomyocytes and smooth muscle cells and were negative for endocardial, myocardial, and smooth muscle markers (arrowheads, *Figure 3B′*, *C′*, and *D′*). Performing wall thickness measurements (see Supplementary material online, *Figure S3F*) and aligning this to the myocardial-arterial boundary confirmed our observations (*Figure 3E*). Between 54 and 70 hpf, the cell numbers in these valve primordia increased (see Supplementary material online, *Figure S4D* and *D′*) and this was associated with cells undergoing division, as demonstrated by incorporation of BrdU (see Supplementary material online, *Figure S4E* and *E′*), occurring equally in both primordia (see Supplementary material online, *Figure S4E′*). Thus, as in mouse and human, the arterial valve forms in conjunction with the arterial root at the myocardial-smooth muscle (myocardial-arterial) boundary (*Figure 3E*).

As positioning of the arterial valve primordia is intimately linked with the transition zone,^15,17^ we next examined whether this region was present in the arterial pole of the zebrafish embryonic heart. As with other vertebrates, zebrafish SHF addition requires the conserved Islet family of transcription factors,^13,40^ with Isl1+, Isl2a+, and Isl2b+ cells present at the arterial pole; however, only *isl2b* is required for cell addition.^40^ At both 46 hpf (*Figure 3F–F″*) and 54 hpf (*Figure 3G–G″*), we clearly identified both endocardial and myocardial cells at the arterial pole of the heart that were Isl1/2+, confirming previous studies that the OFT is SHF derived.^15,29,41–43^ In particular, cardiac troponin+, Isl1/2+ cells confirmed the presence of the transition zone^17^ in the zebrafish OFT (*Figure 3H*). At 54 hpf, cells within the developing valve primordia were also Isl1/2+ and positioned adjacent to the transition zone (*Figure 3G′*, asterisk), defining this as the site of arterial valve formation in zebrafish (*Figure3G″* and *H*).

### 3.4 Arterial valve primordia form through direct differentiation of SHF progenitors

Arterial valve primordia in mice and humans form through two distinct mechanisms: either direct differentiation from SHF progenitors in the ICVSs or via cellularization of ECM-rich cushions by EndoMT-derived cells and neural crest cells.^17,18^

**Figure 4:**
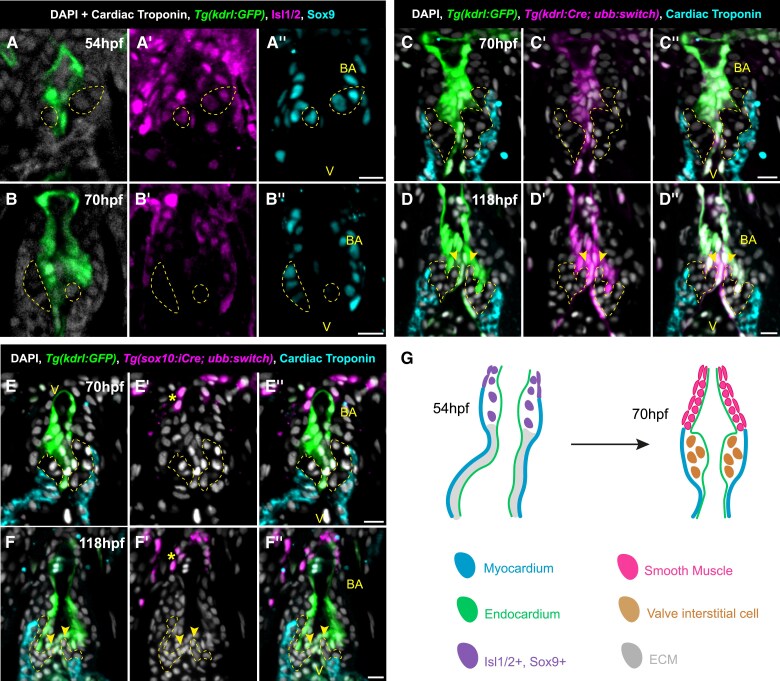
Direct differentiation of SHF progenitors establishes the zebrafish arterial valve. (*A*–*B*″) Representative midline section of immunohistochemistry on *Tg(kdrl:GFP)* (green) embryo at 54 hpf (*A*–*A*″) and 70 hpf (*B*–*B*″) for cardiac troponin (white, membrane), DAPI (white, nuclear) (*A* and *B*), Islet1/2 (Isl1/2, magenta) (*A*′ and *B*′), and Sox9 (cyan) (*A*″ and *B*″). (*A*–*A*″) At 54 hpf, cells of the distal OFT, including within the arterial valve primordia (yellow) co-express Isl1/2 and Sox9 (*n* = 19). (*B*–*B*″) By 70 hpf, cells within the arterial valve primordia (yellow) have down-regulated Isl1/2 (*B*′) but maintain expression of Sox9 (*B*″) defining them as VICs (*n* = 12). (*C*–*D*″) Representative midline sections of lineage tracing of endothelial cells in the OFT of *Tg(kdrl:GFP); Tg(kdrl:Cre); Tg(-3.5ubb:loxP-EGFP-loxP-mCherry)* embryos (magenta) at 70 hpf (*C*–*C*″) and 118 hpf (*D*–*D*″). (*C*–*C*″) Cells of the arterial valve primordia at 70 hpf (yellow), identified between distal-most myocardium (cyan) and endocardium (green), are not of endocardial origin (*n* = 16). (*D*–*D*″) At 118 hpf following primordia remodelling, there is no recombination observed in the arterial valve leaflets (yellow, arrowheads denote tips of leaflets) (*n* = 14). (*E*–*F*″) Representative midline sections of lineage tracing of neural crest cells in the OFT of *Tg(kdrl:GFP); Tg(sox10:iCre, cryaa:DsRed2); Tg(-3.5ubb:loxP-EGFP-loxP-mCherry)* embryos (magenta) at 70 hpf (*E*–*E*″) and 118 hpf (*F*–*F*″). (*E*–*E*″) Cells of the arterial valve primordia at 70 hpf (yellow), identified between distal-most myocardium (cyan) and endocardium (green), are not of neural crest origin (*n* = 12/14). (*F*–*F*″) At 118 hpf following primordia remodelling, there is no recombination observed in the arterial valve leaflets (yellow) (*n* = 8/12, arrowheads denote tips of leaflets). At both stages, recombination is present in the distal tip of the bulbus arteriosus (*E*′ and *F*′, asterisk). (*G*) Summary of expression data from (*A–F*). V, ventricle; BA, bulbus arteriosus. Scale bars: 10 μm.

As we had identified the presence of the transition zone in the zebrafish OFT (*Figure 3F–H*), the development of which is linked to the formation of the ICVSs,^19^ we first examined the expression patterns of Isl1/2 and Sox9 in the arterial valve primordia, as co-expression of these genes is the hallmark of the direct differentiation mechanism.^17^ At 54 hpf, we identified Isl1/2+ Sox9+ cells within the arterial valve primordia (yellow region, *Figure 4A–A″*) as well as distal to the forming valve in the outflow wall that forms the bulbus arteriosus. By 70 hpf, the cells in the primordia had down-regulated Isl1/2 but maintained Sox9 expression (yellow region, *Figure 4B–B″*) as seen in the ICVS of mice.^19^ Cells in the developing bulbus arteriosus were still Isl1/2+ (*Figure 4B′*), indicating later differentiation from SHF, but also co-expressed Sox9 (*Figure 4B″*).

To rule out contributions from the overlying endocardium or neural crest cells to these structures, we used Cre-lox-mediated lineage tracing to identify their contribution. In *Tg(kdrl:Cre); Tg(ubi:loxP-EGFP-loxP-mCherry); Tg(kdrl:GFP)* embryos at both 70 hpf and 118 hpf, we observed recombination of the reporter in the endocardium (*Figure 4C′* and *D′*, magenta), identical to that of *Tg(kdrl:GFP)* (*Figure 4C* and *D*), but no recombination in the cells of the arterial valve primordia (yellow region, *Figure 4C′*; Supplementary material online, *Figure S6A*; *n* = 16/16) or leaflets (yellow region, arrowheads, *Figure 4D″*; Supplementary material online, *Figure S6A*; *n* = 14/14). Conversely, within the same embryos, all cells within the atrioventricular valve showed total recombination (see Supplementary material online, *Figure S6B*–*C″*), confirming that this valve is established by EndoMT^32,42^ in contrast to the arterial valve.

To establish the contribution of neural crest cells to the arterial valve, we generated a new allele of *Tg(sox10:iCre,cryaa:DsRed2)*^44^ (see Supplementary material online, *Figure S5A*–*B′*). In *Tg(sox10:iCre, cryaa:DsRed2); Tg(ubi:loxP-EGFP-loxP-mCherry); Tg(kdrl:GFP)* embryos at 70 hpf, we could identify a neural crest cell contribution to the ventral aorta and distal-most portion of the bulbus arteriosus^40^ (yellow asterisk, *Figure 4E′* and *E″*). In the arterial valve primordia at 70 hpf, we observed no recombination in 12/14 embryos (yellow region, *Figure 4E* and *E′*; Supplementary material online, *Figure S6D*). In the remaining two embryos, we observed a single cell labelled, although the positioning of these cells was not consistent. At 118 hpf, recombination was still observed distally^45^ (*Figure 4F′* and *F″*, asterisk; Supplementary material online, *Figure S5C–C″′*), with 8/12 embryos had no recombination in the arterial valve leaflets (*Figure 4F′* and *F″*; Supplementary material online, *Figure S6D*). In the remaining four embryos, between one and three Cre+ cells were observed in the arterial valve leaflets but had no reproducible spatial localization. Between 70 and 118 hpf, the average percentage of neural crest cells did not increase (see Supplementary material online, *Figure S6D*; 70 hpf: 1.02%, 118 hpf: 2.47%). Neural crest cells were completely absent from the atrioventricular valve in all embryos at 70 hpf (see Supplementary material online, *Figure S6E–E″*; *n* = 14/14). At 118 hpf, we observed Cre+ cells in the leaflets of the atrioventricular valves in 6/10 embryos, supporting previous observations^46^ (see Supplementary material online, *Figure S6F–F″*). There was no correlation between the presence of Cre+ cells in the arterial valve and atrioventricular valve.

Together, these lineage tracing experiments demonstrate that the origin of the arterial valve primordia is EndoMT- and neural crest cell-independent and that the mechanism of primordia formation is direct SHF differentiation, as seen in the two ICVSs of mouse and human.^19,20^ In summary, the formation of the arterial valve primordia occurs at the transition zone, through direct differentiation of SHF progenitors into Sox9+ VICs (*Figure 4G*); defining arterial valve establishment in zebrafish to be a distinct mechanism from that of atrioventricular valve development.

### 3.5 Arterial valve primordia cells are distinct from smooth muscle cells

**Figure 5:**
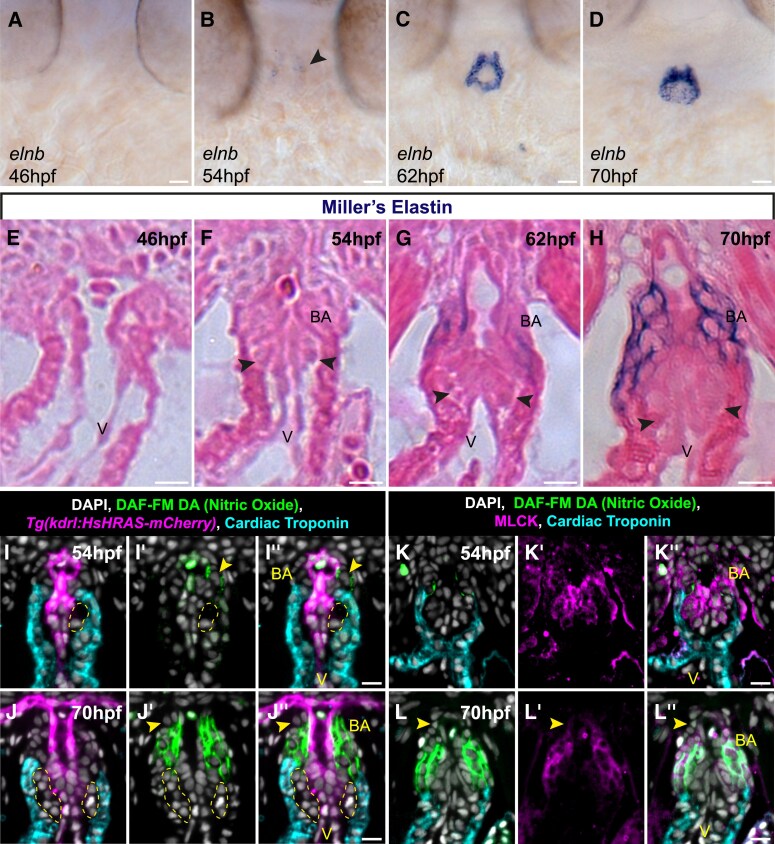
Arterial valve primordia cells are distinct from smooth muscle cells. (*A–D*) Representative mRNA *in situ* hybridization analysis of *elastin b (elnb)* expression during arterial valve primordia formation. *elnb* is not expressed at 46 hpf (*A*) (*n* = 17) and initiates at 54 hpf (*B*, arrowhead) (*n* = 17), and the domain expands between 62 and 70 hpf (*C–D*) (62 hpf, *n* = 17; 70 hpf, *n* = 17). (*E–H*) Midline resin sections of embryos stained for elastin fibres by Miller’s elastin between 46 and 70 hpf. No elastin fibres are present at 46–54 hpf (*E* and *F*) (46 hpf, *n* = 13; 54 hpf, *n* = 12). Fibres are present at 62 hpf (*G*) (*n* = 11), and by 70 hpf (*H*, *n* = 12), the majority of the bulbus arteriosus has elastin fibres, but these are not present in the primordia (arrowheads in *F*–*H*). (*I*–*J*″) Representative midline resin section of immunohistochemistry on *Tg(kdrl:HsHRAS-mCherry)* (magenta) embryos incubated with the NO sensor DAF-FM (green) and stained with cardiac troponin (cyan) and DAPI (white) at 54 hpf (*I*–*I*″) and 70 hpf (*J*–*J*″). At 54 hpf, there is weak, sparse DAF-FM staining of the putative SHF-derived smooth muscle (*n* = 13/18), and there is no staining in the arterial primordia (yellow region). At 70 hpf (*J*–*J*″), DAF-FM staining is present throughout the majority of the bulbus arteriosus, with some cells negative at the distal-most point. There is no staining of the arterial valve primordia (yellow region) (*n* = 13). (*K*–*L*″) Representative midline resin section of immunohistochemistry on embryos incubated with the NO sensor DAF-FM (green) and stained with cardiac troponin (cyan), MLCK (magenta), and DAPI (white) at 54 hpf (*K*–*K*″) and 70 hpf (*L*–*L*″). As in (*I*′), at 54 hpf, there is weak, sparse DAF-FM staining of the putative SHF-derived smooth muscle and no staining in the arterial primordia (yellow region) (*n* = 11/14). In all embryos, MLCK signal is present in the valve primordia and undifferentiated SHF. At 70 hpf (*L*–*L*″), MLCK signal is membrane restricted, absent from the arterial primordia and marks the entirety of the bulbus arteriosus (arrowhead), overlapping with DAF-FM staining (*n* = 14). V, ventricle; BA, bulbus arteriosus. Scale bars: 10 μm.

It has been previously suggested that cells of the arterial valve primordia are smooth muscle, based on analysis of elastin localization.^28^ However, in our analyses, cells within the arterial valve primordia did not express the classical smooth muscle marker Tagln (*Figure 3B′–D′*). To clarify this, we examined the spatio-temporal expression of *elastin b* (*elnb*), by wholemount mRNA *in situ* hybridization. This revealed expression in the distal outflow from 54 hpf onwards, earlier than previously described^47^ (*Figure 5A–D*) coinciding with primordia formation (*Figures 2* and *3*; Supplementary material online, *Figure S3*) and smooth muscle appearance in the bulbus arteriosus (*Figure 3B–D′*). Midline sections in *Tg(kdrl:GFP)* embryos at 54 and 70 hpf revealed no *elnb* expression in cells of the valve primordia (yellow region, Supplementary material online, *Figure S7A*–*B″*). To further confirm this, we performed Miller’s elastin stain at embryonic stages, identifying mature elastin was detectable in the bulbus arteriosus from 62 hpf onwards, but was consistently absent from the valve primordia (arrowheads, *Figure 5E–H*).

Nitric oxide (NO) is a conserved marker of the arterial pole, identified by the sensor DAF-FM,^28,36^ and reported to mark the bulbus arteriosus from 2 dpf onwards.^28,30^ To integrate these observations with our model of OFT development, we examined DAF-FM localization during arterial primordia formation. At 54 hpf, we observed sparse DAF-FM staining in the undifferentiated smooth muscle distal to the myocardium, with no signal in the VICs (*Figure 5I–I″*). By 70 hpf, DAF-FM staining was present throughout most of the bulbus arteriosus, with the absence of staining in the most distal cells (*Figure 5J′ and J″*, arrowhead). No DAF-FM staining was observed in the valve primordia (*Figure 5J–J″*, yellow region).

We also examined co-localization of DAF-FM with a second smooth muscle marker, myosin light chain kinase (MLCK),^45^ reported to be expressed in the BA from 3 dpf.^28,48^ At 54 hpf, we observed diffuse MLCK signal throughout the distal OFT, complementary to cardiac troponin that extended into the valve primordia (*Figure 5K–K″*). By 70 hpf, MLCK expression was more clearly membrane-restricted in the smooth muscle that was distal to the myocardium (*Figure 5L′*), and similar to Isl1/2, MLCK expression in the primordia of the arterial valve was down-regulated between 54 and 70 hpf (*Figure 5K′* and *L′*). DAF-FM overlapped with MLCK; however, the MLCK expression extended more distally into the connection between the bulbus arteriosus and ventral aorta (*Figure 5L–L″*, arrowhead).

Finally, our analyses suggested that both VICs of the arterial valve primordia and smooth muscle cells of the bulbus arteriousus are Sox9+ (*Figure 4B″*), similar to what has been observed in the mouse aorta.^24^ We confirmed this at 54 hpf, where Sox9 overlapped with MLCK throughout the distal OFT (yellow region, Supplementary material online, *Figure S7C–C″*), and at 70 hpf, where Sox9 was present in both MLCK+ smooth muscle and the now MLCK- cells of the arterial valve primordia (see Supplementary material online, *Figure S7C′* and *D′*).

Together, this confirms that the SHF-derived cells of the arterial valve primordia are distinct from the smooth muscle that resides distally and, together with our genetic lineage tracing, demonstrates a high level of evolutionary conservation in arterial pole development.

### 3.6 Loss of SHF progenitors disrupts arterial valve primordia development

To further support the role of the SHF in establishing the arterial valve primordia, we examined OFT development in a mutant where SHF addition is known to be disrupted. The highly conserved transcription factor *tbx1* is required to maintain the SHF progenitor pool,^49,50^ and loss of *TBX1* results in 22q11.2 deletion syndrome (DiGeorge syndrome).^51^*tbx1* mutant zebrafish have been shown to have a shorter OFT,^29,38,49^ but the impact on the arterial valve remains uninvestigated.

**Figure 6:**
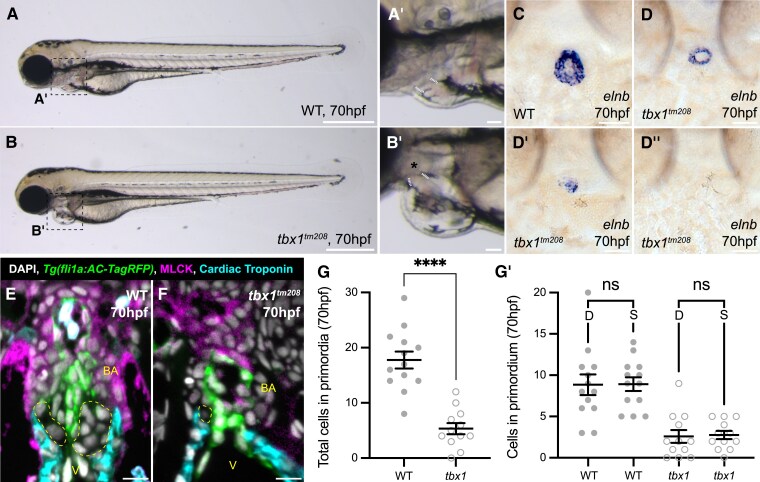
*tbx1* mutants have a dysmorphic OFT and hypocellular arterial valve primordia. (*A*–*B*′) Representative brightfield image of WT sibling (*A*, *n* = 6) and *tbx1^tm208^* homozygous mutant (*B*, *n* = 12) at 70 hpf, dashed boxes in (*A*) and (*B*) highlights heart shown in (*A*′) and (*B*′). In *tbx1^tm208^* mutants (*B*′), the pharyngeal arches are absent (asterisk), and the angle of the OFT angle is steeper than in WT (white). (*C*–*D*″) Representative images of mRNA *in situ* hybridization for *elnb* in WT sibling (*C*) and *tbx1^tm208^* homozygous mutants (*D*–*D*″) at 70 hpf. *tbx1* mutants display variability in size of *elnb* domain. (*E* and *F*) Representative midline sections of immunohistochemistry on WT sibling (*E*) and *tbx1^tm208^* homozygous mutants (*F*) carrying *Tg(fli1a:AC-TagRFP)* to mark the endocardium (green), cardiac troponin (cyan), and MLCK (magenta). The *tbx1^tm208^* OFT is smaller and dysmorphic with diffuse MLCK expression. (*G* and *G*′) Quantification of number of cells in arterial valve primordia (yellow, *E* and *F*) at 70 hpf in WT sibling and *tbx1^tm208^* homozygous mutants at 70 hpf. Loss of *tbx1* results in a significant reduction in number of VICs and impacts the dextral (D) and sinistral (S) primordia equally (WT sibling, *n* = 13; *tbx1^tm208^*, *n* = 12). (*G*) and (*G*′): Mean ± S.E.M, Welch’s unpaired *t*-tests. ****, *P* < 0.0001; ns, not significant. V, ventricle; BA, bulbus arteriosus; D, dextral; S, sinistral. Scale bars: (*A*) and (*B*): 500 μm; (*A*′) and (*B*′): 50 μm; (*C*)–(*D*′): 20 μm; (*E*) and (*F*): 10 μm.

At 70 hpf, *tbx1* mutants had fully penetrant pericardial oedema, absence of pharyngeal arches, and an abnormally positioned OFT (*Figure6A*–*B′*). Using *elnb* to mark the bulbus arteriosus revealed that this structure was either severely hypoplastic (*Figure 6D* and *D′*) or absent (*Figure 6D″*) in *tbx1* mutants. Whilst WT siblings had a regular, pear-shaped aortic root housing the arterial valve primordia (*Figure 6E*), *tbx1* mutants had a grossly disorganized OFT (*Figure 6F*). Expression of MLCK in WT siblings was robust and membrane-restricted, enabling VICs to be defined by their absence of myocardial, endocardial, or smooth muscle marker expression (yellow region, *Figure 6E*). In *tbx1* mutants, MLCK expression was often totally absent from the OFT and showed variable expression between individual embryos, likely reflecting the spectrum of OFT hypoplasia identified by analysis of *elnb* expression (*Figure 6D–D″*). This prevented identification of VICs by absence of MLCK expression; therefore, as before, we defined VICs to be within the myocardial collar, under the endocardium, but negative for both of these markers (*Figures 3–5*; Supplementary material online, *Figures S4* and *S6*). This approach identified a significant reduction in the number of VICs, with both primordia impacted similarly (*Figure 6G* and *G′*), supporting that the arterial valve primordia are formed from SHF progenitors.

### 3.7 Conserved requirement for Wnt-PCP signalling in OFT formation

**Figure 7:**
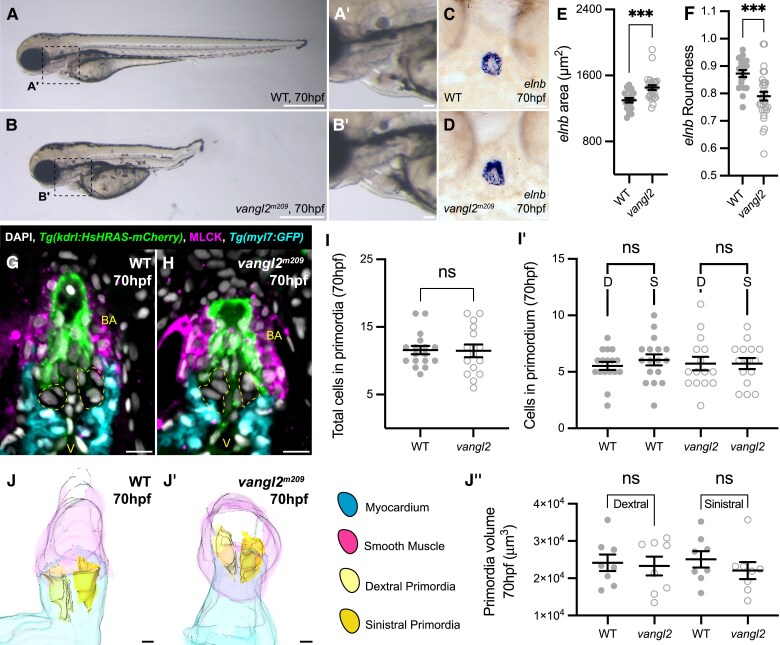
*vangl2* mutants have misshapen OFT. (*A*–*B*′) Representative brightfield image of WT sibling (*A*, *n* = 7) and *vangl2^m209^* homozygous mutant (*B*, *n* = 15) at 70 hpf, dashed boxes in (*A*) and (*B*) highlights heart shown in (*A*′) and (*B*′). The overt morphology of the *vangl2^m209^* OFT appears normal (white). (*C* and *D*) Representative images of mRNA *in situ* hybridization for *elnb* in sibling (*C*) and *vangl2^m209^* homozygous mutant (*D*) at 70 hpf. (*E*) Quantification of area of *elnb* domain at 70 hpf. Loss of *vangl2* results in a larger *elnb* domain. (*F*) Quantification of roundness of *elnb* domain at 70 hpf. Loss of *vangl2* leads to a less round *elnb* domain (WT sibling, *n* = 19; *vangl2^m209^*, *n* = 28). (*G* and *H*) Representative midline sections of immunohistochemistry on WT sibling (*G*) and *vangl2^m209^* homozygous mutants (*H*) carrying *Tg(kdrl:HsHRAS-mCherry)* to mark the endocardium (green) and *Tg(myl7:GFP)* to mark the myocardium (cyan) and MLCK (magenta). The *vangl2* OFT appears squat, with the myocardial collar less pronounced. (*I* and *I*′) Quantification of number of cells in arterial valve primordia (yellow) at 70 hpf in WT sibling and *vangl2^m209^* homozygous mutants at 70 hpf. Loss of *vangl2* does not impact number of VICs ((WT sibling, *n* = 17; *vangl2^m209^*, *n* = 15), D, dextral primordia; S, sinistral primordia). (*J*–*J*″) Quantification of arterial valve primordia volume by 3D reconstruction of WT sibling (*J*) and *vangl2^m209^* (*J*′) homozygous mutant OFT from immunohistochemistry. Loss of *vangl2* does not impact primordia volume (*J*″). (*E*), (*F*), (*I*), and (*I*′): Mean ± SEM, Welch’s unpaired *t*-tests. (*J*″): Mean ± SEM, Brown–Forsyth and Welch’s ANOVA. ***, *P* < 0.001; ns, not significant. V, ventricle; BA, bulbus arteriosus; D, dextral; S, sinistral. Scale bars: (*A*) and (*B*): 500 μm; (*A*′) and (*B*′): 50 μm; (*C*), (*D*), (*J*), and (*J*′): 20 μm; (*G*) and *H*): 10 μm.

We have previously shown that SHF-specific ablation of the core PCP component *Vangl2* in mice disrupts organization of the transition zone, resulting in smaller or misplaced posterior ICVS, dysplastic aortic leaflets and BAV.^17,19^ As the OFT phenotype of *vangl2* mutant zebrafish has never been characterized, we investigated possible conservation of Vangl2 function. At 70 hpf, the *vangl2* mutant zebrafish had a shorter antero-posterior axis (*Figure 7A* and *B*), but overtly, heart morphology appeared normal (*Figure 7A′* and *B′*). Analysis of *elnb* expression identified that the bulbus arteriosus was significantly larger, but less round in *vangl2* mutants compared to siblings at 70 hpf (*Figure 7C–F*). MLCK expression in *vangl2* mutants was comparable to WT siblings (*Figure 7G* and *H*), and its membrane restriction suggested normal maturation of smooth muscle cells in *vangl2* mutants (*Figure 5K′* and *L′*; Supplementary material online, *Figure S7C*–*D″*). Despite the abnormal shape of the OFT in *vangl2* mutants (*Figure 7C–H*), the primordia were positioned at the myocardial arterial boundary as in WT (*Figure 7G* and *H*) and the number of VIC in the primordia was not different to WT siblings (*Figure 7I–I′*). Three-dimensional reconstruction of the embryonic OFT showed that primordia volume was unchanged between WT siblings and *vangl2* mutants at 70 hpf (*Figure 7J–J′*; *n* = 8 for each genotype), suggesting that the shape of the primordia in *vangl2* mutants could be abnormal. Analysis of the later stages of arterial valve development in *vangl2* mutants was precluded by the formation of pericardial oedema between 3 and 4 dpf (data not shown) that would impact remodelling events non-autonomously.

In summary, we have shown that loss of *tbx1*, a known regulator of SHF development,^29,49^ leads to a small, dysmorphic OFT accompanied by a dramatic loss of cells in the arterial valve primordia, supporting our data that these primordia are SHF-derived. Loss of *vangl2*, a gene known to be required for SHF deployment in mice,^17^ leads to a misshapen OFT, although the primordia are normally populated, suggesting a conserved role for *vangl2* in organization of SHF cell addition during arterial pole development.

## 4. Discussion

**Figure 8:**
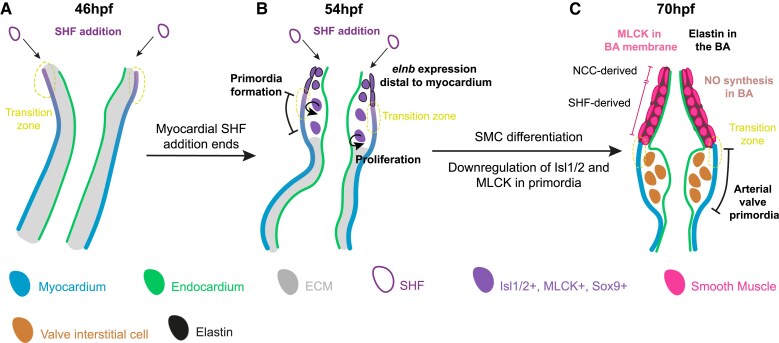
The zebrafish arterial valve forms by direct differentiation of SHF progenitors. Model of zebrafish arterial valve formation. (*A*) At 46 hpf, addition to the OFT from the SHF occurs at the transition zone, where cells co-express Isl1/2 and mature cardiomyocyte markers, before down-regulating Isl1/2. (*B*) At 54 hpf, SHF addition no longer adds myocardium to the OFT, but instead smooth muscle which expresses *elnb*. This switch is the site of formation of the primordia (the transition zone), forming a bulge between the myocardium and endocardium. Cells within the early primordia proliferate and share similar expression patterns to the distal smooth muscle, but do not express *elnb*. (*C*) By 70 hpf, the smooth muscle cells of the bulbus arteriosus are now more molecularly distinct from the cells of the arterial valve primordia. The smooth muscle expresses *elnb*, MLCK, Tagln, and Sox9, is producing NO, and is surrounded by elastin fibres. The interstitial cells of the arterial valve primordia have down-regulated Isl1/2 and MLCK but maintain Sox9. The primordia are devoid of cells expressing *elnb* or any elastin fibres.

In this study, we have characterized the mature structure and embryonic origins of the zebrafish arterial valve. We have shown that both leaflets are established through direct differentiation of SHF progenitors (*Figures 4* and *8*), which is comparable to the development of the anterior and non-coronary leaflets of the mouse pulmonary and aortic valves respectively.^19^ However, it is important to appreciate that this is not a pulmonary, nor aortic valve, as it has been termed in some studies.

There are clear differences between the overall appearance of the arterial valve in zebrafish and mammals. Most obviously, the zebrafish arterial valve is bicuspid (*Figure 1*; Supplementary material online, *Figures S1* and *S2*) with no interleaflet triangles, nor continuity between the arterial and atrioventricular valve (*Figure 1*). There are commissures and valve sinuses, but no coronary ostia as the coronary circulation in fish originates after the gills. Despite these differences, a basal ring, concomitant with the ventriculo-arterial junction, can be seen and the distal attachments of the leaflets define a sino-tubular junction (*Figure 1*). Histological analysis of ECM composition of the valve revealed structures that were comparable to those found in the adult mouse (*Figure 1*; Supplementary material online, *Figure S2*) and in humans. Enriched elastin deposition on the luminal surface of the arterial leaflets is in keeping with the ventricularis, whilst the spongiosa appears present, marked by a proteoglycan-rich ECM. The fibrosa in zebrafish is not well established with only minimal collagen deposition which is present on the luminal aspect of the leaflet.

Subtle differences in ECM content have been reported across vertebrates, with greater collagen and elastin content within the leaflets of larger mammals suggested to be due to the different haemodynamic environments.^10^ Indeed, a recent study has shown that under unidirectional flow which is experienced on the luminal-facing surface of valve leaflets, VICs implement an elastogenic programme.^52^ The relative difference in heart rate^53,54^ may explain the lack of mature elastin in the leaflets of the P90 mouse (*Figure 1*; Supplementary material online, *Figure S2*)^52,55^ despite presence in both human and zebrafish. Furthermore, there may also be a cell lineage-specific contribution, where the absence of a clear fibrosa layer in zebrafish may be due to the lack of neural crest cells populating the valve primordium (*Figure 4*). Together, it is tempting to speculate that the formation of the scalloped basal ring, interleaflet triangles, and perhaps collagen of the fibrosa may be secondary either to the mechanical effects of a high-pressure cardiovascular system, complete septation of the OFT, or both. Overall, we identified a general and conserved, localization pattern of ECM components in the zebrafish aortic root (*Figure 1*; Supplementary material online, *Figure S2*), and this model may prove more useful than mouse in understanding the mechanism of elastogenesis in vertebrate arterial valve leaflets.^56^

In zebrafish, both arterial valve primordia arise directly from undifferentiated SHF at the most distal extent of the OFT transition zone, with no contribution from neural crest cells or EndoMT (*Figures 4* and *8*; Supplementary material online, *Figure S6*). As in the mouse, we observed no trans-differentiation from myocardium and ruled out a smooth muscle identity of these cells (*Figures 3* and *5*; Supplementary material online, *Figure S7*). The conservation of direct differentiation of VICs from SHF progenitors, from fish to mammals, suggests a relation to the ancestral mechanism of arterial valve development, predating EndoMT in the OFT cushions and the co-option of neural crest for complete OFT septation. The presence of the transition zone in zebrafish (*Figure 3*), chick,^57^ mouse,^17,19^ human,^16^ and probably *Xenopus*,^58^ a domain that is tightly linked with the site of valve primordia formation, supports this further. In *Xenopus*, neural crest cells are not required for the incomplete septation of the OFT; instead, these cells remain in the aortic sac and associated arches.^59^ This contribution is very similar to what we and others have observed in zebrafish, where the neural crest forms part of the ventral aorta (*Figure 4*; Supplementary material online, *Figure S5*).^40,45^ Altogether, this demonstrates that the OFT of zebrafish is composed of SHF-derived myocardium proximally, above this, SHF-derived smooth muscle and, most distally, neural crest-derived smooth muscle (*Figures 4* and *8*; Supplementary material online, *Figure S7*). These contributions from SHF and neural crest cells again show a high level of conservation with the mouse OFT; SHF generates the smooth muscle at the base of the aorta and pulmonary trunk, whilst the smooth muscle of the ascending aorta and associated arteries is of neural crest origin.^8,60^

Our work clarifies the developmental origins of the arterial valve and demonstrates definitively that it is distinct from the atrioventricular valve (*Figure 4*; Supplementary material online, *Figure S6*). During our lineage tracing, we identified that sox10+ presumptive neural crest cells are not present in the atrioventricular valve before 70 hpf, expanding a previous study which identified their presence at 5 dpf, which we confirmed^46^ (see Supplementary material online, *Figure S6*). We observed a minimal and variable contribution of sox10+ cells to the arterial valve at both 70 and 118 hpf (*Figure 4*; Supplementary material online, *Figure S6*). It is unclear exactly what role these cells play in either valve, particularly given their inconsistent spatial location and sudden appearance in the atrioventricular valve at 118 hpf. A possible mechanism, supported by mouse studies, may be that rather than originating from the neural crest; these cells have up-regulated *sox10* in the developing valves once they have begun to form.^61–63^

Multiple studies have suggested conservation of the zebrafish arterial pole development and anatomy^15,27,28,38,64^ which we have expanded. We have identified that *elnb* is the earliest, exclusive marker of the bulbus arteriosus and that MLCK marks both the bulbus arteriosus and VICs at 54 hpf (*Figure 5*). As this smooth muscle differentiates, Transgelin begins to be expressed, MLCK becomes membrane restricted, and NO is produced (marked by DAF-FM). Interestingly, at the same time, the VICs of the primordia down-regulate MLCK, possibly suggesting a shared developmental origin of these two populations of cells. We also observed strong Sox9 signal present in the smooth muscle of the OFT and other structures in the pharyngeal region (*Figure 4*; Supplementary material online, *Figure S7*, and data not shown). In P1 mice, phosphorylated Sox9 is present in the wall of the root, supporting that Sox9 expression in the OFT wall is in fact a conserved pattern.^24^

The analysis of *vangl2* and *tbx1* zebrafish mutants provides important links between development of the zebrafish and mammalian OFTs (*Figures 6* and *7*). Loss of *Tbx1* in mice results in a shortened distal OFT with development of the anterior ICVS impacted,^19,65^ and we have identified comparable phenotypes in the zebrafish *tbx1* mutant (*Figure 6*), mirroring the congenital heart malformations seen in 22q11.2 deletion syndrome (DiGeorge). However, whilst the anterior ICVS is affected in *Tbx1* mice mutants due to its requirement for posterior SHF addition,^65,66^ both arterial leaflets were impacted similarly in the zebrafish *tbx1* mutant (*Figure 6*), suggesting that the unseptated OFT may not have the distinct regional identities observed in mouse.^67^ In contrast, loss of *vangl2* resulted in a more subtle defect, with sufficient SHF-derived cells present in the primordia that were positioned normally, but the overall size and shape of the smooth muscle domain marked by *elnb* were abnormal (*Figure 7*). This suggests that similar to mouse,^17^ loss of PCP signalling may lead to disordered cell addition to the arterial pole, rather than loss as observed in *tbx1* mutants. Little is known about homozygous human *VANGL2* mutations as these are likely to be incompatible with development.^68^

Disrupted aortic valve morphology in patients is associated with altered haemodynamics and aortopathies, yet which abnormality (flow, valve structure or wall integrity) is the driver of the pathology remains unanswered.^69–71^ Zebrafish are uniquely placed to investigate these interactions, due to high conservation of flow-dependent processes and accessibility for repeated *in vivo* live imaging. Previous studies have shown that cardiac function is required for the growth of the OFT^29,39^ and *tnnt2a* morphants (which lack a heartbeat) are reported to have a complete absence of arterial valve primordia.^30^ Additionally, it has been recently shown that disrupted TGF-β signalling in zebrafish results in dilatation of the arterial pole that is transcriptionally similar to Marfan syndrome and thoracic aortic aneurysm dissection.^72^ This, together with our description of the developmental conservation of OFT structure, suggests the possibility of zebrafish as a model to investigate a wider repertoire of arterial pole malformations.

There are numerous benefits to studying valvulogenesis in zebrafish: temporal reproducibility of the process, accessibility of the key stages, external development for live imaging, and that this is all possible prior to reaching the stage at which studies become regulated (typically 5 dpf). Whilst previously, the majority of developmental studies on valve developmental has occurred in mice and then shown to be relevant in humans and other species such as zebrafish, it is now apparent that more developmental work can be done principally in zebrafish and confirmed in mice, greatly reducing the use of mammals in fundamental research.

In summary, we have shown that the two leaflets of the zebrafish arterial valve are established by the same developmental mechanism as the mouse and human intercalated leaflets, distinct to that of atrioventricular valve formation. This opens up new avenues for using zebrafish in understanding human congenital heart disease through functional testing of human gene variants.

**Translational perspective**

Large genomic studies of patients with bicuspid aortic valve (BAV) have identified numerous variants predicted to be causative. However, due to a lack of suitable, *in vivo* functional assays, these variants are rarely tested. This means that discussion of risk to family members and prognosis is not yet widely possible. Here, we show that zebrafish demonstrate a high level of conservation in arterial valve development with the intercalated leaflets in human, establishing zebrafish as a suitable *in vivo* model for subtypes of BAV that can begin to overcome the disconnect between clinical genetics and developmental biology.

## Supplementary Material

cvae230_Supplementary_Data

## Data Availability

All data that support the findings not presented in main figures and supplementary files are available from the corresponding author upon request.
