# Supplementary material for: Zebrafish arterial valve development occurs through direct differentiation of second heart field progenitors
Source: Cardiovasc Res. 2024 Oct 26;121(1):157–73. doi: 10.1093/cvr/cvae230 (PMC11998914; doi:10.1093/cvr/cvae230)
Supplement: cvae230_Supplementary_Data [file cvae230_supplementary_data.zip › Derrick_CVR-2024-0342_Supplemental.pdf]

Zebrafish arterial valve development occurs through direct differentiation of second heart field progenitors

Christopher J. Derrick, Lorraine Eley, Ahlam Alqahtani, Deborah J. Henderson, Bill Chaudhry*

Supplemental Figures 1-7

Movies 1-4

Supplemental Methods

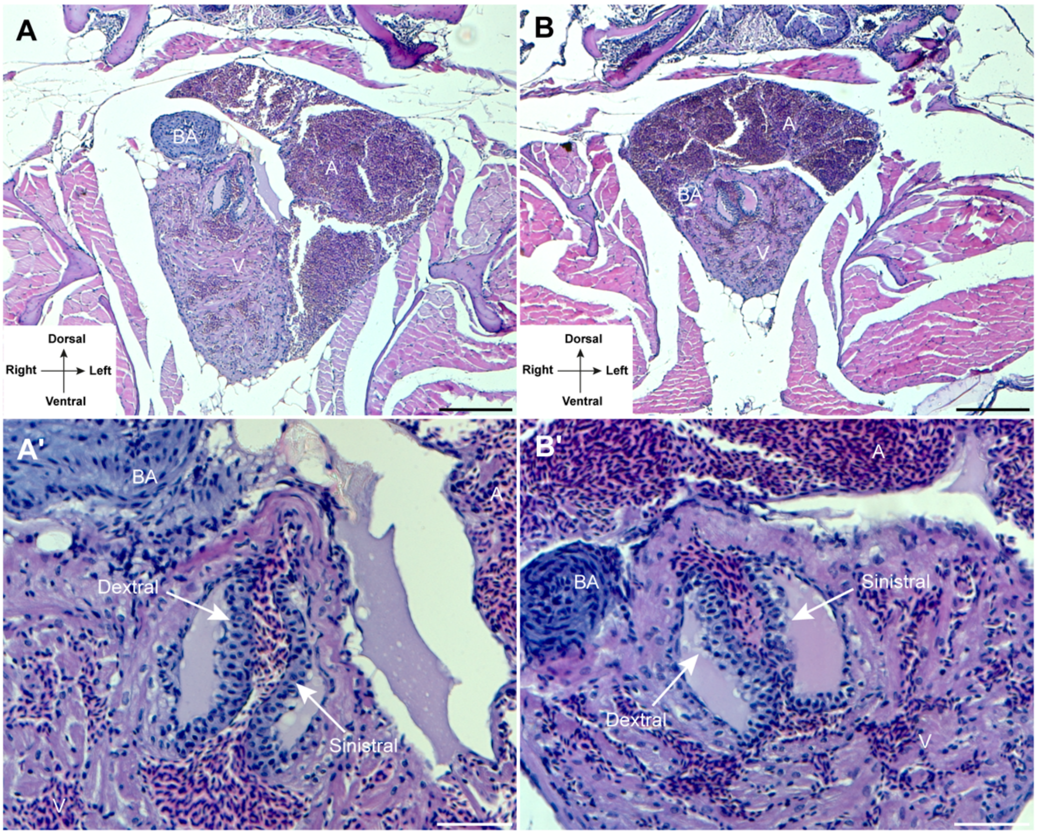

Supplemental Figure 1. Zebrafish arterial valve leaflets are positioned laterally. (A-A') Anterior view of 117dpf (Standard Length: 2.28cm) female zebrafish stained with Haematoxylin and Eosin showing the position of heart within the body cavity (A) and the orientation of the leaflets (A'). **(B-B')** Anterior view of 117dpf (Standard Length: 1.88cm) male zebrafish stained with Haematoxylin and Eosin showing the position of heart within the body cavity (B) and the orientation of the leaflets (B'). The line of apposition of the arterial valve leaflets is along the antero-posterior axis, and the leaflets are predominantly located on the left (sinistral) and right (dextral). V: Ventricle, A: Atrium, BA: Bulbus Arteriosus. Dorsal: up. Scale bars: A, B: 200μm, A', B': 50μm.

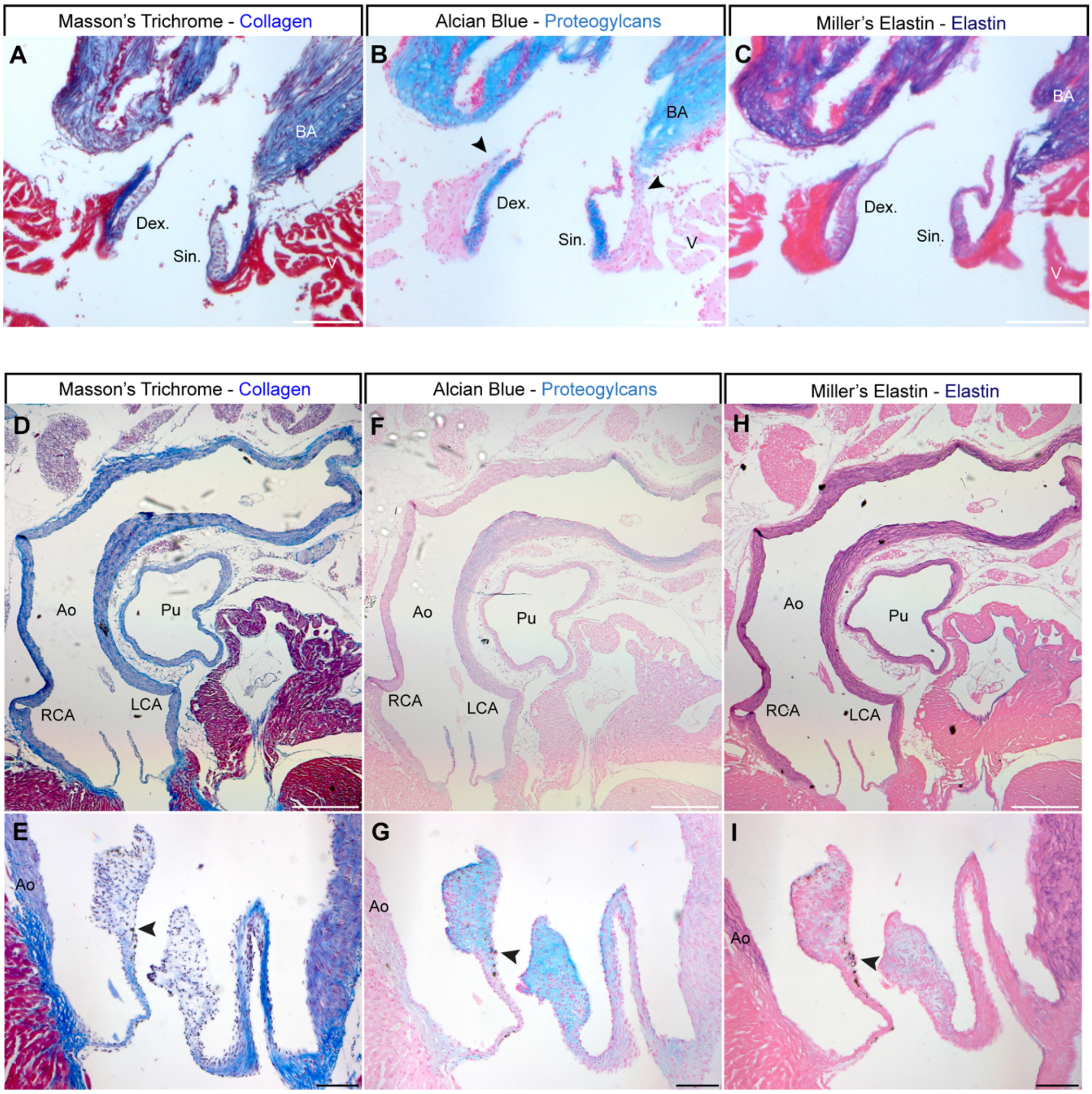

Supplemental Figure 2. Localisation of ECM components in the adult zebrafish and mouse arterial pole.

(A) Masson's Trichrome stained longitudinal section of the adult zebrafish arterial pole (wide field view of Fig. 1H). Collagen is present in the bulbus arterious and continuous with the arterial root. **(B)** Alcian Blue stained longitudinal section of the adult zebrafish arterial pole (wide field view of Fig. 1K). The bulbus arteriosus is rich in sulphated proteoglycans, with some staining present in the distal-most part of the arterial root (arrowheads). **(C)** Miller's Elastin stained longitudinal section of the adult

zebrafish arterial pole (wide field view of Fig. 1N). Elastin is abundant in the bulbus arteriosus and continuous with the arterial root. **(D)** Masson's Trichrome stained longitudinal section of the arterial pole of a P90 mouse (widefield view of Fig. 1P). Collagen is present in the great vessels and the root of the aortic valve. **(E)** Oblique cut of left and right aortic valve leaflets of P90 mouse (same as Fig. 1P-Q) stained with Masson's Trichrome. Collagen is present in the root, hinge and base of the leaflets and more strongly than in the wall of the aorta. **(F)** Alcian Blue stained longitudinal section of the arterial pole of a P90 mouse (wide field view of Fig. 1S). Sulfated proteoglycans are present at low levels in the great vessels. **(G)** Oblique cut of left and right aortic valve leaflets of P90 mouse (same as Fig. 1S-T) stained with Alcian Blue. Sulfated proteoglycans are found concentrated in the tips of the leaflets in a reciprocal pattern to Collagen and present at low levels in the hinge. Very little stain is present in the wall of the aorta. **(H)** Miller's Elastin-stained longitudinal section of the arterial pole of a P90 mouse (wide field view of Fig. 1V). Mature elastin fibres are clearly visible in the wall of the aorta and pulmonary artery. **(I)** Oblique cut of left and right aortic valve leaflets of P90 mouse (same as Fig. 1V-W) stained with Miller's Elastin. Mature elastic fibres are present in the wall of the aorta, with diffuse staining in the tips likely reflecting immature elastin. Elastin is absent from the hinge and base of the leaflets. Neural-crest derived melanocytes are observable in some sections (arrowheads, E, G, I). BA: Bulbus Arteriosus, V: Ventricle, Dex: Dextral leaflet, Sin: Sinistral leaflet, Ao: Aorta, Pu: Pulmonary artery, RCA: Right coronary artery, LCA: Left coronary artery. Anterior: up. Scale bars: A-C: 100μm. D, F, H: 500μm. E, G, I: 50μm.

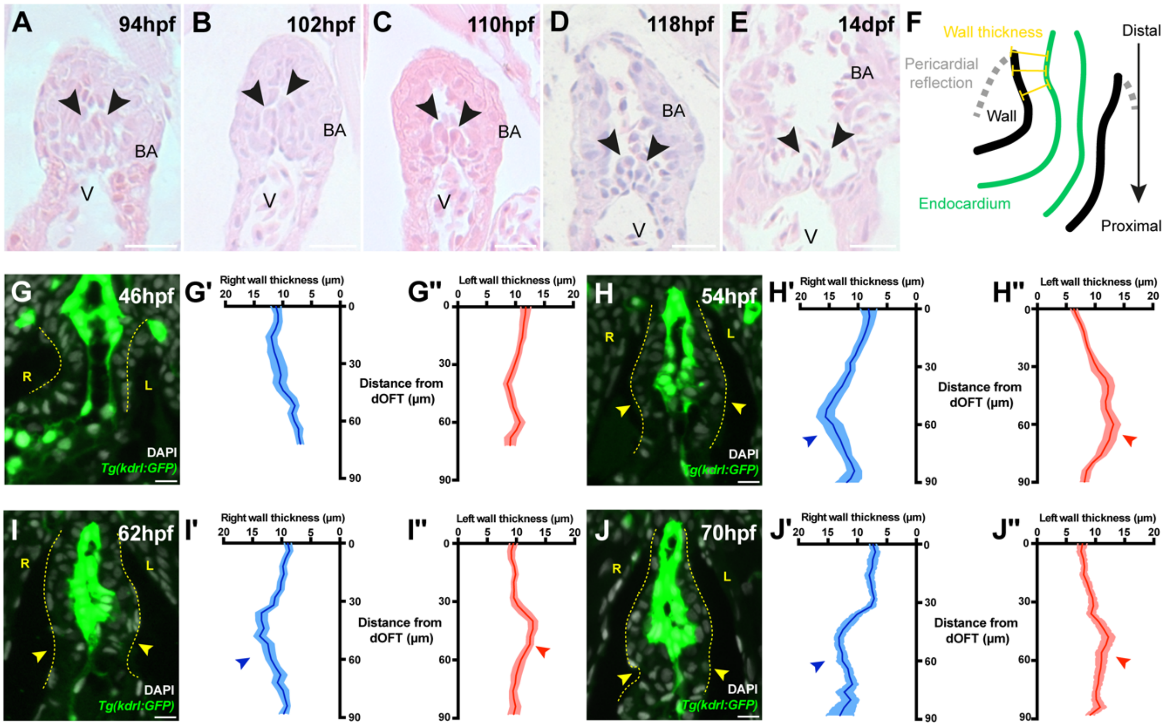

Supplemental Figure 3. Arterial valve primordia formation occurs between 2-3dpf.

(A-E) Representative Haematoxylin and Eosin-stained midline section along the long axis of the OFT at 94hpf (A) (n=5), 102hpf (B) (n=6), 110hpf (C) (n=5), 118hpf (D) (n=5) and 14dpf (E) (n=5). Two leaflets are visible (arrowheads), which have begun to remodel between 5-14dpf and appear thinner. **(F)** Schematic of approach to measure wall thickness. The perpendicular distance (yellow) between the inner edge of the endocardium (marked by *Tg(kdrl:GFP)*, green) and the outer edge of the wall (black) is measured with the pericardial reflection (grey) defined as the distal most point of the OFT. **(G-G")** Quantification of wall thickness at 46hpf. No primordia are present at this stage. Yellow line denotes edge of wall (n=11). **(H-H")** Quantification of wall thickness at 54hpf. Increased wall thickness, representing arterial primordia are visible. Yellow line denotes edge of wall, yellow arrowheads in (H) denote primordia in (H'-H'') (n=8). **(I-I")** Quantification of wall thickness at 62hpf.

Increased wall thickness, representing arterial primordia are visible. Yellow line denotes edge of wall, yellow arrowheads in (I) denote primordia in (I'-I'') (n=11). **(J-J'')** Quantification of wall thickness at 70hpf. Increased wall thickness, representing arterial primordia are visible. Yellow line denotes edge of wall, yellow arrowheads in (J) denote primordia in (J'-J'') (n=10). V: Ventricle, dOFT: distal outflow tract, R: morphological right, L: morphological left. Anterior: up. G-J: thickness is Mean ± S.E.M. Scale bars: A-E: 20μm, G-J: 10μm.

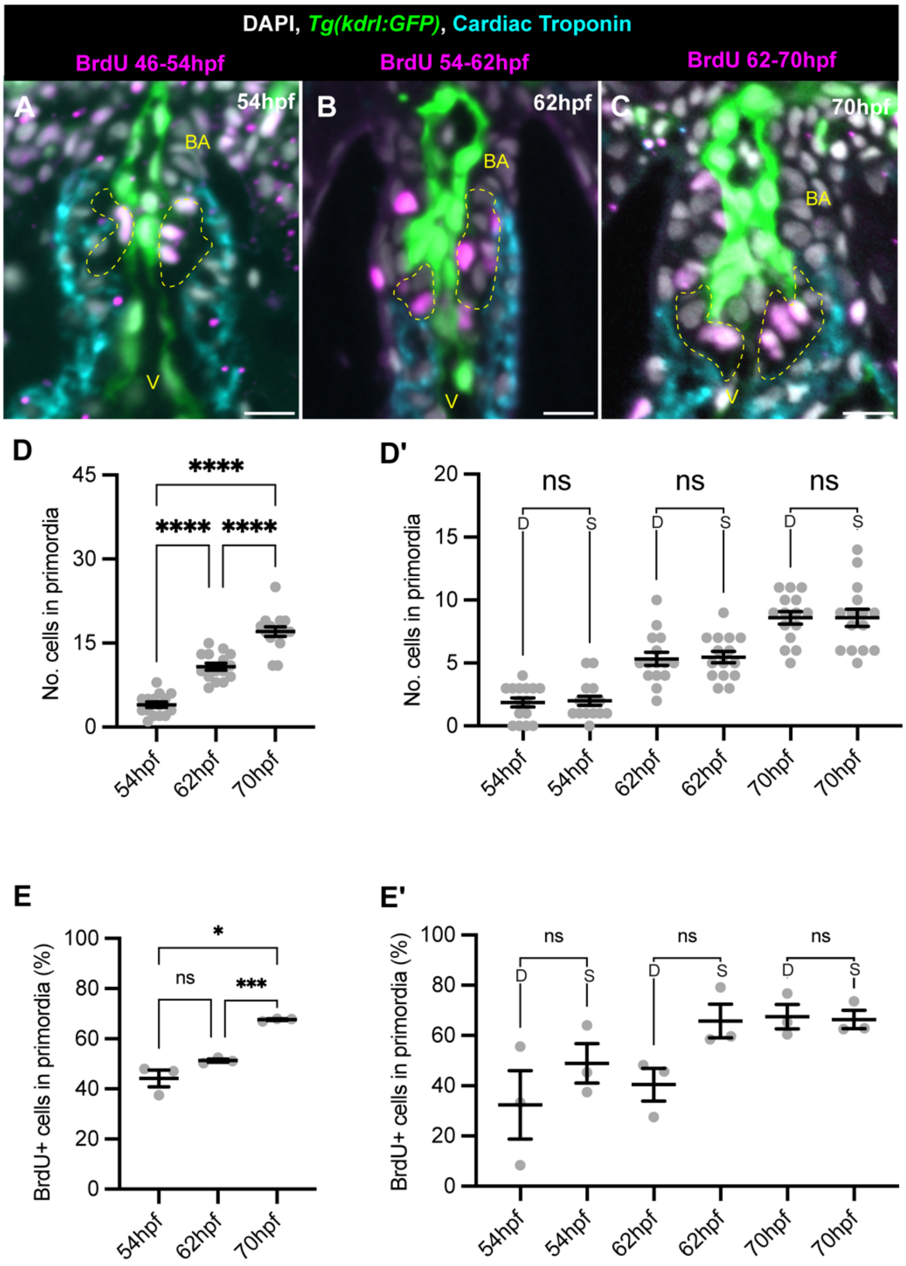

Supplemental 4. Proliferation in the arterial valve primordia accompanies increase in cell number

(A) Representative midline resin section of *Tg(kdrl:GFP)* (green) embryo incubated in BrdU between 46-54hpf, fixed at 54hpf and stained for BrdU (magenta) and Cardiac Troponin (cyan). Region of quantification (primordia) denoted by yellow lines. **(B)** Representative midline resin section of *Tg(kdrl:GFP)* embryo incubated in BrdU between 54-62hpf, fixed at 62hpf and stained for BrdU and Cardiac Troponin.

Region of quantification (primordia) denoted by yellow lines. **(C)** Representative midline resin section of *Tg(kdrl:GFP)* embryo incubated in BrdU between 62-70hpf, fixed at 70hpf and stained for BrdU and Cardiac Troponin. Region of quantification (primordia) denoted by yellow lines. **(D)** Quantification of total cell number in the arterial valve primordia between 54-70hpf, cell number significantly increases over the course of development. **(D')** Breakdown of quantification into dextral (D) and sinistral (S) primordia, there are no differences between the left and right primordia (N=3, 5 fish per N, n=15). (E) Quantification of percentage of BrdU positive cells in the arterial valve primordia between following BrdU pulses, levels of proliferation increase significantly between 62hpf and 70hpf. **(E')** Breakdown of quantification in (E) into dextral (D) and sinistral (S) there are no differences between the left and right primordia (N=3, 5 fish per N). V: Ventricle, BA: Bulbus Arteriosus. Scale bars: 10µm. D-E': Mean ± S.E.M, Brown-Forsythe and Welch ANOVA with multiple comparisons. *: $p<0.05$, ***: $p<0.001$, ****: $p<0.0001$, ns: not significant.

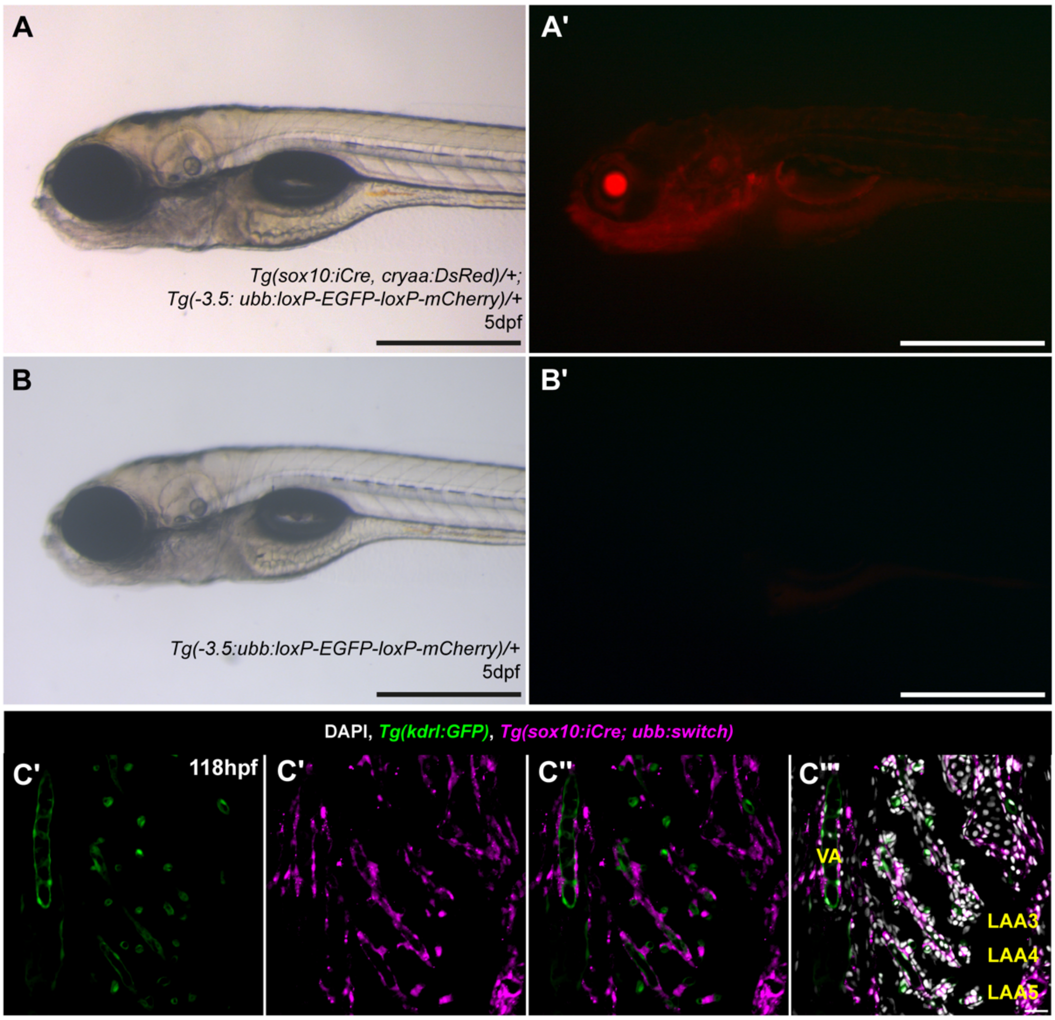

Supplemental Figure 5. Characterisation of *Tg(sox10:iCre, cryaa:DsRed2)*^{n6}

(A) Brightfield image of live *Tg(sox10:iCre, cryaa:DsRed2)*/+; *Tg(-3.5:ubb:loxP-EGFP-loxP-mCherry)/*+ at 5dpf. **(A')** Same fish from (A) viewed under fluorescence with DsRed filter. Recombination is present in neural crest-derived structures (craniofacial). Signal in the eye is the transgenesis marker. **(B)** Brightfield image of live *Tg(-3.5:ubb:loxP-EGFP-loxP-mCherry)*/+ at 5dpf. **(B')** Same fish from (B) viewed under fluorescence with DsRed filter. No recombination is visible. **(C-C''')** Representative section of left aortic arches and ventral aorta of *Tg(kdrl:GFP)/+; Tg(sox10:iCre, cryaa:DsRed2)*/+; *Tg(-3.5:ubb:loxP-EGFP-loxP-mCherry)/*+ at 118hpf (5dpf), the endocardium (green) is surrounded by vascular smooth muscle

cells of neural crest origin (magenta). VA: Ventral Aorta, LAA: Left Aortic Arch. Scale bars: A-B’: 500μm, C-C’’’: 20μm.

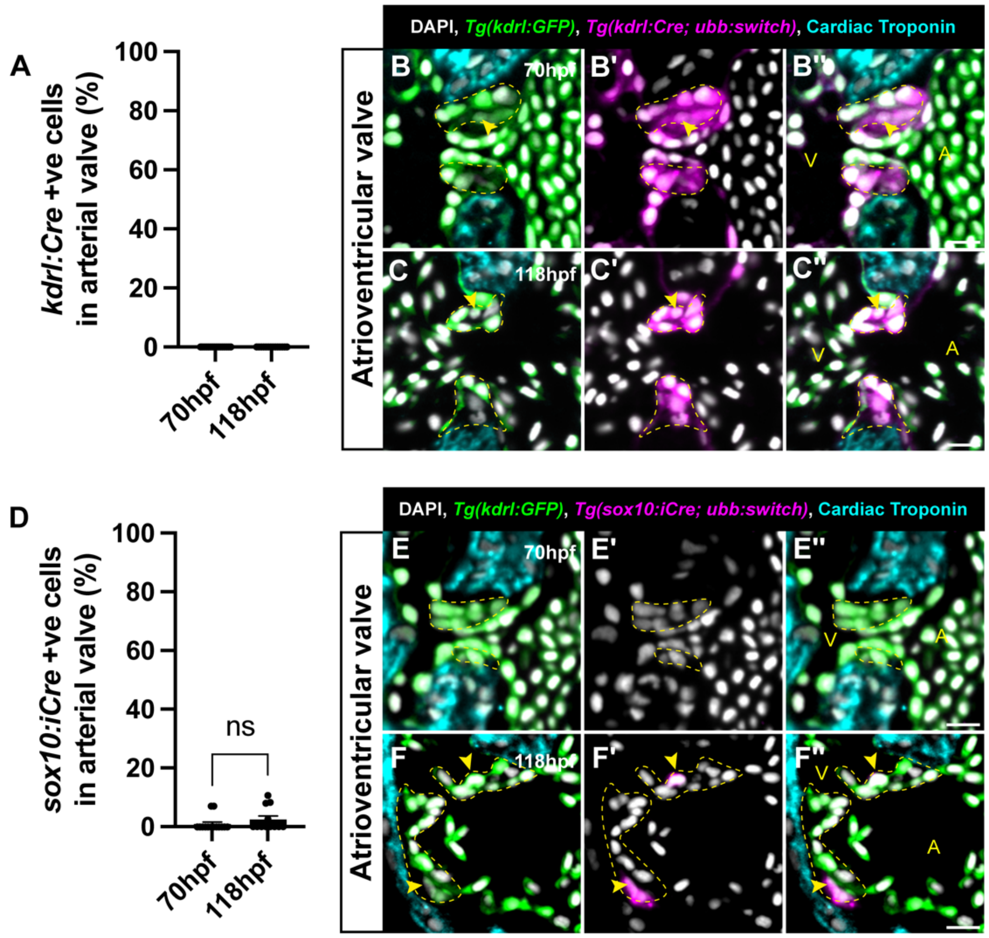

Supplemental Figure 6. Distinct origins of valve primordia in zebrafish

(A) Quantification of percentage of kdrl:Cre positive cells in the arterial valve at 70hpf and 118hpf (see Fig 4C-D”). No cells of endothelial origin contribute to the valve (70hpf: n=16, 118hpf: n=14). **(B-C”)** Representative examples of lineage tracing of endothelial cells in the atrioventricular valve of *Tg(kdrl:GFP); Tg(kdrl:Cre); Tg(-3.5ubb:loxP-EGFP-loxP-mCherry)* embryos, stained for cardiac troponin (cyan) at 70hpf (B-B”) and 118hpf (C-C”). (B-B”) At 70hpf, cells of the superior and inferior leaflets (yellow) are kdrl:GFP (green) and kdrl:Cre (magenta) positive. (C-C”) By 118hpf, cells in the intersitium of the atrioventricular leaflets (yellow) are no longer kdrl:GFP positive, but lineage tracing confirms that they originated from kdrl+ cells (yellow arrowhead). **(D)** Quantification of percentage of sox10:iCre positive cells in

the arterial valve at 70hpf and 118hpf (see Fig 4E-F”). At 70hpf, 2/14 embryos have cells in the arterial valve primordia (one cell each, mean percentage contribution to the valve primordia: 1.02%). At 118hpf, 4/12 embryos have cells in the arterial valve (one with one, one with two and two with three, mean contribution to the valve primordia: 2.47%). There is no significant increase in the number of sox10:iCre cells that contribute to the arterial valve between 70hpf and 118hpf. **(E-F”)** Representative examples of lineage tracing of endothelial cells in the atrioventricular valve of *Tg(kdrl:GFP); Tg(sox10:iCre, cryaa:DsRed2); Tg(-3.5ubb:loxP-EGFP-loxP-mCherry)* embryos, stained for cardiac troponin at 70hpf (E-E”) and 118hpf (F-F”). (E-E”) At 70hpf, there is no contribution to the atrioventricular leaflets (yellow) (n= 14). (F-F”) At 118hpf, 6/10 embryos have cells (arrowheads) contributing to the atrioventricular valve leaflets (yellow). B: Mann-Whitney test, ns: not significant. B-C, E-F anterior: up, left: right. V: Ventricle, A: Atrium. Scale bar: 10μm

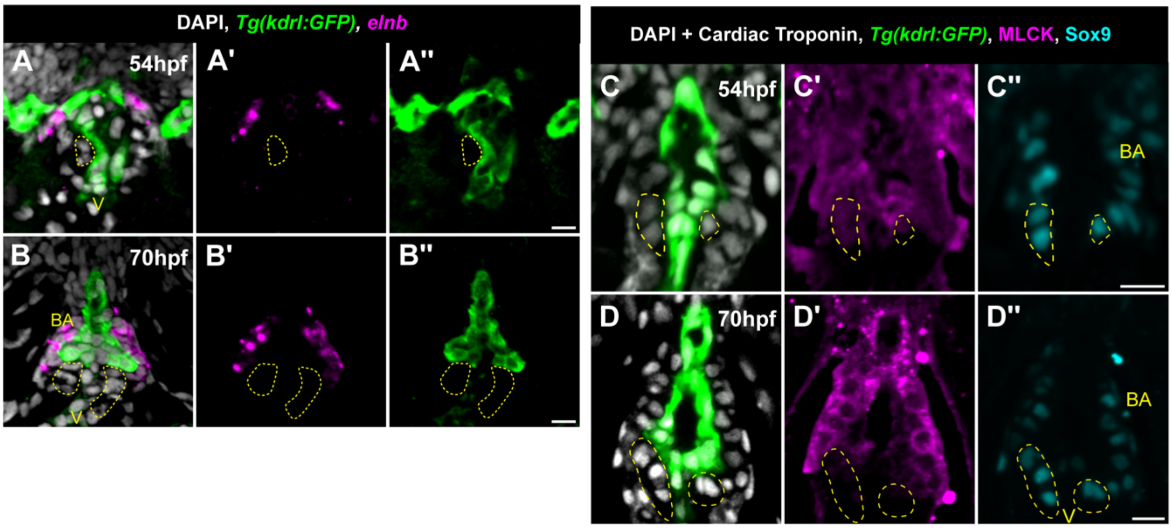

Supplemental Figure 7. Arterial valve primordia are distinct from smooth muscle cells.

(A-A") Midline section of fluorescent mRNA *in situ* hybridisation of *elnb* (magenta, A') in *Tg(kdrl:GFP)* (green, A") embryo at 54hpf counterstained with DAPI (white). *elnb* is not expressed in the cells of the primordia (yellow) but is expressed at the distal-most aspect of the OFT (n=7). **(B-B")** Midline section of fluorescent mRNA *in situ* hybridisation in of *elnb* (magenta, B') in *Tg(kdrl:GFP)* (green, B") embryo at 70hpf counterstained with DAPI (white). *elnb* is not expressed in the cells of the primordia (yellow) but is expressed at the distal-most aspect of the OFT (n=7). **(C-C")** Representative midline section of immunohistochemistry on *Tg(kdrl:GFP)* (green) embryo at 54hpf for Cardiac Troponin (white, membrane), DAPI (white, nuclear) (C), Myosin Light Chain Kinase (MLCK, magenta) (C') and Sox9 (cyan) (C"). Cells distal to the myocardium, including those in the arterial valve primordia (yellow) show diffuse MLCK expression and co-express Sox9 (n=19). **(D-D")** Representative midline section of immunohistochemistry on *Tg(kdrl:GFP)* embryo at 70hpf. MLCK expression in the bulbus arteriosus is restricted to the membrane and complementary to Cardiac Troponin. Cells within the arterial valve primordia (yellow) have downregulated MLCK (G') but maintain expression of Sox9 (G") (n=13). V: Ventricle, BA: Bulbus Arteriosus. Scale bar: 10μm

Movie 1. Arterial valve primordia at 62hpf

Representative movie of *Tg(kdrl:GFP)* at 62hpf (Widefield view of Fig 2I) at half live speed. Two primordia are visible between the Ventricle and Bulbus Arteriosus (n=13). BA: Bulbus Arteriosus, V: Ventricle, A: Atrium.

Movie 2. Arterial valve primordia at 70hpf

Representative movie of *Tg(kdrl:GFP)* at 70hpf (Widefield view of Fig 2J) at half live speed. Two primordia are visible between the Ventricle and Bulbus Arteriosus (n=14). BA: Bulbus Arteriosus, V: Ventricle, A: Atrium.

Movie 3. Arterial valve primordia at 78hpf

Representative movie of *Tg(kdrl:GFP)* at 78hpf (Widefield view of Fig 2K) at half live speed. Sinuses have begun to form in the two primordia are visible between the Ventricle and Bulbus Arteriosus (n=11/15). BA: Bulbus Arteriosus, V: Ventricle, A: Atrium.

Movie 4. Arterial valve primordia at 86hpf

Representative movie of *Tg(kdrl:GFP)* at 86hpf (Widefield view of Fig 2L) at half live speed. Sinuses have formed in the two primordia, defining the leaflets of the arterial valve between the Ventricle and Bulbus Arteriosus (n=17/17). BA: Bulbus Arteriosus, V: Ventricle, A: Atrium.

Supplemental Methods

Zebrafish lines and general husbandry

The following previously described lines were used: *Tg(kdrl:GFP)*^{la116} (1), *Tg(kdrl:Cre)*^{s898} (2), *Tg(-3.5:ubb:loxP-EGFP-loxP-mCherry)*^{cz1701} (3), *Tg(fli1a:AC-TagRFP)*^{sh511} (4), *Tg(myl7:EGFP)*^{twu34} (5), *Tg(kdrl:HsHRAS-mCherry)*^{s916} (6), *tbx1*^{tm208} (7–9), *vangl2*^{m209} (9,10). *Tg(kdrl:GFP)*^{la116} was maintained in a Capser (*roy*$^{a9/a9}$*; mitf*$^{aw2/aw2}$) background (11), apart from Cre lineage tracing (AB, Casper out-cross), all other lines were maintained in a mixed AB background. *tbx1*^{tm208} genotyping was carried out as previously described (8). *vangl2*^{m209} genotyping was carried out as previously described (12). *Tg(kdrl:Cre)*^{s898} was maintained by breeding Cre+ males with WT(AB) females (13,14).

Embryos were obtained from natural pairwise mating of adults, maintained at 28.5ºC in E3 medium (5 mM NaCl, 0.17 mM KCl, 0.33 mM $CaCl_2$, 0.33 mM $MgSO_4$) and staged according to Kimmel 1995 (15). Where necessary, between 22-24 hpf, embryos were transferred into E3 medium containing 0.003% 1-phenyl-2-thiourea (PTU, Sigma P7629) to prevent pigment formation, aiding imaging. Embryos were fixed in 4% Paraformaldehyde (PFA, P6148 Sigma) in 1x Phosphate Buffered Saline (PBS, Oxoid BR0014G) overnight at 4ºC. The next day embryos were washed in PBST (1xPBS with 0.2% Tween (Sigma P2287)) and serially dehydrated to 100% MeOH and stored at -20ºC or washed in PBS and serially dehydrated to 70% EtOH and stored at room temperature. For mutant analyses, embryos were rinsed from fixative into PBST, the caudal fin taken for gDNA extraction and genotyping as above. Once genotyped, WT siblings and mutant embryos were collated and dehydrated separately.

Collection of adult zebrafish tissue

Adult zebrafish hearts were manually dissected following terminal anaesthesia using Tricaine methanesulfonate (MS222, Merck E10521) and destruction of the brain. Hearts were briefly incubated in room temperature Ringer's solution with 10mM Calcium Chloride (116mM NaCl, 2.9mM KCl, 10mM $CaCl_2$, 5mM HEPES), before overnight fixation in 4% PFA 4ºC. Hearts were washed three times in PBS at room temperature, and then serially dehydrated to 70% EtOH for long term storage at room temperature.

Adult zebrafish were culled by terminal anaesthesia (MS222, Merck E10521) and destruction of the brain, with images taken prior to fixation for measurement of standard length (16). Whole fish were fixed at in 4% PFA at 4C for 4 nights, washed 3 times in PBS at room temperature, decalcified in 0.5M EDTA pH8.0 (Merck, 27285) with agitation at 4ºC for 4 nights, and then serially dehydrated to 70% EtOH for long term storage at room temperature.

Establishment of *Tg(sox10:iCre, cryaa:DsRed2)* line

The previously described *sox10:iCre,cryaa:DsRed2* plasmid (17) was injected together with *tol2* mRNA (18) and Phenol Red (Sigma, P0290) into the yolk of 1-cell stage embryos obtained from in-crossing WT(AB) adults. Each embryo was injected with 33.3pg of plasmid, 33.3pg of *tol2* mRNA and 25% Phenol Red. Healthy F0 embryos were screened at 4dpf for the presence of DsRed2+ eyes (marker of transgenesis) to enrich for plasmid integration and raised to adulthood. Adult F0 were outcrossed to WT(AB), embryos with DsRed+ eyes at 4dpf were raised to adulthood (F1). A single F1 adult male was selected to establish the line at F2, based on 50% transmission of DsRed+ eyes at 4dpf (representing a single insertion). All experiments were carried out using at least F3. The line is designated *Tg(sox10:iCre, cryaa:DsRed2)*^{n6} and is maintained by breeding Cre+ males with WT(AB) females (13,14).

Lineage tracing

Adult male heterozygous *Tg(kdrl:Cre)*^{s898} or *Tg(sox10:iCre, cryaa:DsRed2)*^{n6} were outcrossed to *Tg(-3.5:ubb:loxP-EGFP-loxP-mCherry)*$^{cz170/+}$ *Tg(kdrl:GFP)*$^{la116/+}$ females to ensure tissue specificity of Cre activity (13,14). Embryos positive for both GFP transgenes were fixed at 70hpf, washed three times in PBST and genotyped for Cre. *Tg(kdrl:Cre)*^{s898} using primers Forward: 5'- AACGAGTGATGAGGTTCGCAAGAACC-3' and Reverse: 5'-AAATCAGTGCGTTCGAACGCTAGAG-3' with GoTaq G2 (Promega): 35 cycles, 30s extension time, 55C annealing temperature. *Tg(sox10:iCre, cryaa:DsRed2)*^{n6} using primers Forward: 5'- GCAGCCACACCATTCTTTCT-3' and Reverse: 5'-GAAGACCATCCAACAGCACC-3' with GoTaq G2: 35 cycles, 30s extension time, 58ºC annealing temperature. Embryos were split into Cre+ve and Cre-ve and serial dehydrated to 100% MeOH at -20ºC. Cre-ve embryos showed no recombination (data not shown).

mRNA *in situ* hybridisation

For chromogenic mRNA *in situ* hybridisation, embryos were fixed overnight in 4% paraformaldehyde (PFA, Cell Signalling Technology 12606) and steps were carried out as previously described (19). Fluorescent mRNA *in situ* was performed using the APExBIO Tyr-Cy3 TSA Kit (Stratech, K1051-APE) and carried out as previously described (20), the *Tg(kdrl:GFP)* signal was boosted post-ISH by immunohistochemistry as described above, but with only 30 mins of blocking. FISH samples were rapidly dehydrated before resin embedding.

Resin embedding of zebrafish embryos

For resin sectioning, embryos and adult hearts were dehydrated in 100% EtOH (this is performed rapidly for embryos with fluorescent staining, see (21)). Tissue was moved from

EtOH into Hardener 1 (Technovit 8100), allowed to settle and then rinsed twice in Hardener 1, and incubated at 4ºC for at least 2hrs. For embedding, Hardener 2 was mixed with fresh Hardener 1 according to manufacturer's instructions, tissue was transferred into H1-H2 mix, oriented and sealed in an airtight container at 4ºC overnight. Where necessary, blocks were cut and re-embedded for specific orientations.

Adult hearts were cut at 7µm sections and embryos cut at 4µm sections using a RMC MT990 Microtome. H&E of resin sections was carried out as previously described (21) and allowed to dry. H&E and embryonic Miller's-stained slides were mounted in Histomount. Resin sections of immunohistochemistry where mounted in 1:1 mix PBS-Vectashield with DAPI (Vector labs H-1200), or where Cardiac Troponin was revealed using Alexa350, 1:1 PBS-Vectashield (Vector labs H-1000) with VectaShield with DAPI diluted to 0.3%.

Quantification of wall thickness

Midline sections of embryos, where a lumen is visible extending from the ventricle to the connection of the heart with the surrounding mesoderm were selected. In Fiji (22,23), using an adapted add_mark_every_x_microns.ijm macro (https://gist.github.com/mutterer/95cc6f027080674af68ca65fae8d1051) the length of the left and right *Tg(kdrl:GFP)* signal at the distal most point of the heart (defined as 0 for measurements) was marked and subdivided into 4µm sections. Using the DAPI channel, the outer surface of the wall of the heart was traced using the freehand line tool and the perpendicular distance from the endocardium to the outer edge of the wall was measured. The myo-arterial boundary was defined as the first 4µm subdivision of the *Tg(kdrl:GFP)* signal in which the wall was positive for Cardiac Troponin signal. For embryos where more than one section was measured, an average of the wall thickness across the sections was taken.

Quantification of primordia cell number, proliferation, OFT length and *elnb* domain

In Fiji, sections through the OFT were selected on the basis of a clear lumen with continuous endocardial transgene signal from the distal-most point of the OFT to the ventricle. Using the myocardial signal, a straight line was drawn across the distal-most signal – defining the arterial primordia as cells below this line that were present between the myocardium and endocardium but did not express either of these markers. Cells were counted in alternate sections.

For BrdU analysis, cells were defined as BrdU+ or BrdU- within the arterial valve primordia (as defined above). For each individual embryo (n), average BrdU+ cells in the primordia were quantified (as above). A single average was calculated from the 5 embryos of one treatment group (N), this was repeated twice, giving 3 experimental replicates, each consisting of 5 embryos.

The shape of the *elnb* signal was traced using the freehand selection tool in Fiji and shape parameters measured. Embryos were genotyped post image acquisition.

Supplemental References

1. Choi J, Dong L, Ahn J, Dao D, Hammerschmidt M, Chen J-N. FoxH1 negatively modulates flk1 gene expression and vascular formation in zebrafish. Dev Biol. 2007 Apr 15;304(2):735–44.

2. Bertrand JY, Chi NC, Santoso B, Teng S, Stainier DYR, Traver D. Haematopoietic stem cells derive directly from aortic endothelium during development. Nature. 2010 Mar 4;464(7285):108–11.

3. Mosimann C, Kaufman CK, Li P, Pugach EK, Tamplin OJ, Zon LI. Ubiquitous transgene expression and Cre-based recombination driven by the ubiquitin promoter in zebrafish. Development. 2011 Jan;138(1):169–77.

4. Savage AM, Kurusamy S, Chen Y, Jiang Z, Chhabria K, MacDonald RB, et al. tmem33 is essential for VEGF-mediated endothelial calcium oscillations and angiogenesis. Nat Commun. 2019 Feb 13;10(1):732.

5. Huang C-J, Tu C-T, Hsiao C-D, Hsieh F-J, Tsai H-J. Germ-line transmission of a myocardium-specific GFP transgene reveals critical regulatory elements in the cardiac myosin light chain 2 promoter of zebrafish. Dev Dyn. 2003 Sep;228(1):30–40.

6. Hogan BM, Bos FL, Bussmann J, Witte M, Chi NC, Duckers HJ, et al. Ccbe1 is required for embryonic lymphangiogenesis and venous sprouting. Nat Genet. 2009 Apr;41(4):396–8.

7. Piotrowski T, Schilling TF, Brand M, Jiang YJ, Heisenberg CP, Beuchle D, et al. Jaw and branchial arch mutants in zebrafish II: anterior arches and cartilage differentiation. Development. 1996 Dec;123:345–56.

8. Piotrowski T, Ahn D, Schilling TF, Nair S, Ruvinsky I, Geisler R, et al. The zebrafish van gogh mutation disrupts tbx1, which is involved in the DiGeorge deletion syndrome in humans. Development. 2003 Oct;130(20):5043–52.

9. Haffter P, Granato M, Brand M, Mullins MC, Hammerschmidt M, Kane DA, et al. The identification of genes with unique and essential functions in the development of the zebrafish, Danio rerio. Development. 1996 Dec;123:1–36.

10. Jessen JR, Topczewski J, Bingham S, Sepich DS, Marlow F, Chandrasekhar A, et al. Zebrafish trilobite identifies new roles for Strabismus in gastrulation and neuronal movements. Nat Cell Biol. 2002 Aug;4(8):610–5.

11. White RM, Sessa A, Burke C, Bowman T, LeBlanc J, Ceol C, et al. Transparent adult zebrafish as a tool for in vivo transplantation analysis. Cell Stem Cell. 2008 Feb 7;2(2):183–9.

12. Derrick CJ, Szenker-Ravi E, Santos-Ledo A, Alqahtani A, Yusof A, Eley L, et al. Functional analysis of germline VANGL2 variants using rescue assays of vangl2 knockout zebrafish. Hum Mol Genet. 2024 Jan 7;33(2):150–69.

13. Hans S, Kaslin J, Freudenreich D, Brand M. Temporally-controlled site-specific recombination in zebrafish. PLoS ONE. 2009 Feb 27;4(2):e4640.

14. Carney TJ, Mosimann C. Switch and trace: recombinase genetics in zebrafish. Trends Genet. 2018 May;34(5):362–78.

15. Kimmel CB, Ballard WW, Kimmel SR, Ullmann B, Schilling TF. Stages of embryonic development of the zebrafish. Dev Dyn. 1995 Jul 1;203(3):253–310.

16. Parichy DM, Elizondo MR, Mills MG, Gordon TN, Engeszer RE. Normal table of postembryonic zebrafish development: staging by externally visible anatomy of the living fish. Dev Dyn. 2009 Dec;238(12):2975–3015.

17. Abdul-Wajid S, Demarest BL, Yost HJ. Loss of embryonic neural crest derived cardiomyocytes causes adult onset hypertrophic cardiomyopathy in zebrafish. Nat Commun. 2018 Nov 2;9(1):4603.

18. Kwan KM, Fujimoto E, Grabher C, Mangum BD, Hardy ME, Campbell DS, et al. The Tol2kit: a multisite gateway-based construction kit for Tol2 transposon transgenesis constructs. Dev Dyn. 2007 Nov 1;236(11):3088–99.

19. Thisse C, Thisse B. High-resolution in situ hybridization to whole-mount zebrafish embryos. Nat Protoc. 2008;3(1):59–69.

20. Welten MCM, de Haan SB, van den Boogert N, Noordermeer JN, Lamers GEM, Spaink HP, et al. ZebraFISH: fluorescent in situ hybridization protocol and three-dimensional imaging of gene expression patterns. Zebrafish. 2006;3(4):465–76.

21. Sullivan-Brown J, Bisher ME, Burdine RD. Embedding, serial sectioning and staining of zebrafish embryos using JB-4 resin. Nat Protoc. 2011 Jan;6(1):46–55.

22. Schindelin J, Arganda-Carreras I, Frise E, Kaynig V, Longair M, Pietzsch T, et al. Fiji: an open-source platform for biological-image analysis. Nat Methods. 2012 Jun 28;9(7):676–82.

23. Mutterer J, Zinck E. Quick-and-clean article figures with FigureJ. J Microsc. 2013 Oct;252(1):89–91.
